# Molecular Atlas of Postnatal Mouse Heart Development

**DOI:** 10.1161/JAHA.118.010378

**Published:** 2018-10-14

**Authors:** Virpi Talman, Jaakko Teppo, Päivi Pöhö, Parisa Movahedi, Anu Vaikkinen, S. Tuuli Karhu, Kajetan Trošt, Tommi Suvitaival, Jukka Heikkonen, Tapio Pahikkala, Tapio Kotiaho, Risto Kostiainen, Markku Varjosalo, Heikki Ruskoaho

**Affiliations:** ^1^ Drug Research Program and Division of Pharmacology and Pharmacotherapy Faculty of Pharmacy University of Helsinki Finland; ^2^ Drug Research Program and Division of Pharmaceutical Chemistry and Technology Faculty of Pharmacy University of Helsinki Finland; ^3^ Institute of Biotechnology and HiLIFE Helsinki Institute of Life Science University of Helsinki Finland; ^4^ Department of Future Technologies Faculty of Mathematics and Natural Sciences University of Turku Finland; ^5^ Steno Diabetes Center Copenhagen Gentofte Denmark; ^6^ Department of Chemistry Faculty of Science University of Helsinki Finland

**Keywords:** heart development, heart regeneration, metabolomics, neonatal mouse cardiomyocyte, proteomics, transcriptomics, Developmental biology, Metabolism, Myocardial Regeneration, Physiology, Proteomics

## Abstract

**Background:**

The molecular mechanisms mediating postnatal loss of cardiac regeneration in mammals are not fully understood. We aimed to provide an integrated resource of mRNA, protein, and metabolite changes in the neonatal heart for identification of metabolism‐related mechanisms associated with cardiac regeneration.

**Methods and Results:**

Mouse ventricular tissue samples taken on postnatal day 1 (P01), P04, P09, and P23 were analyzed with RNA sequencing and global proteomics and metabolomics. Gene ontology analysis, KEGG pathway analysis, and fuzzy c‐means clustering were used to identify up‐ or downregulated biological processes and metabolic pathways on all 3 levels, and Ingenuity pathway analysis (Qiagen) was used to identify upstream regulators. Differential expression was observed for 8547 mRNAs and for 1199 of 2285 quantified proteins. Furthermore, 151 metabolites with significant changes were identified. Differentially regulated metabolic pathways include branched chain amino acid degradation (upregulated at P23), fatty acid metabolism (upregulated at P04 and P09; downregulated at P23) as well as the HMGCS (HMG‐CoA [hydroxymethylglutaryl‐coenzyme A] synthase)–mediated mevalonate pathway and ketogenesis (transiently activated). Pharmacological inhibition of HMGCS in primary neonatal cardiomyocytes reduced the percentage of BrdU‐positive cardiomyocytes, providing evidence that the mevalonate and ketogenesis routes may participate in regulating the cardiomyocyte cell cycle.

**Conclusions:**

This study is the first systems‐level resource combining data from genomewide transcriptomics with global quantitative proteomics and untargeted metabolomics analyses in the mouse heart throughout the early postnatal period. These integrated data of molecular changes associated with the loss of cardiac regeneration may open up new possibilities for the development of regenerative therapies.

**Clinical Perspective**

**What Is New?**

- This study is the first combining transcriptomics with untargeted proteomic and global metabolomic analyses over several time points in the early postnatal heart and, as such, provides an extensive resource of molecule abundance for future mechanistic studies.
- This report is the first showing temporal regulation of mevalonate and ketone body metabolism in the postnatal heart and identifies their potential roles in the regulation of neonatal cardiomyocyte cell cycle.

**What Are the Clinical Implications?**

- These integrated molecule‐level data may open up new possibilities for the development of regenerative therapies.
- The work highlights the importance of metabolic pathways as potential novel drug targets for cardioregenerative therapeutics.

## Introduction

Adult human hearts possess a negligible regenerative capacity; therefore, cell loss in response to myocardial infarction (MI) leads to scar formation, remodeling of the surrounding myocardium, progressive impairment of cardiac function, and eventually to heart failure (reviewed by Sutton and Sharpe^1^ and Talman and Ruskoaho^2^). Approximately only 0.5% to 1% of human cardiomyocytes are renewed annually through proliferation of existing cardiomyocytes.^3^, ^4^ This is insufficient for regeneration after MI, which can cause a loss of up to 25% (≈1 billion) of cardiomyocytes.^5^ The renewal rate is higher in infants and adolescents than in elderly people, indicating higher regenerative potential in children.^3^, ^6^ Remarkably, certain amphibians and fish as well as neonatal rodents can fully regenerate their hearts after an injury.^7^, ^8^, ^9^ This occurs predominantly through proliferation of remaining cardiomyocytes in the areas adjacent to the injury.^8^, ^10^ However, rodents lose this regenerative capacity within 7 days after birth because of cardiomyocyte cell cycle withdrawal,^8^ which is considered to represent a major hurdle for the development of regeneration‐inducing therapies.^11^, ^12^, ^13^ In line with cardiac regeneration in neonatal rodents, full functional recovery of a newborn baby with a massive MI suggests that humans possess a similar intrinsic capacity to regenerate their hearts at birth.^14^ Consequently, it is crucial to elucidate the molecular mechanisms mediating the postnatal loss of cardiac regeneration.

At birth, the resistance of pulmonary circulation drops dramatically because of the expansion of lung alveoli, causing an increase in systemic blood pressure and shunt closure. This in turn induces a steep increase in arterial blood oxygen content. Increased cardiac workload and altered metabolic environment induce a switch from anaerobic glycolysis, which is the main source of energy in the embryonic heart, to mitochondrial fatty acid β‐oxidation soon after birth.^15^ This switch is controlled by signaling that involves HIF1α (hypoxia‐inducible factor 1α) and HAND1 (heart and neural crest derivatives expressed 1).^16^ At birth, HAND1 expression is rapidly downregulated, allowing cardiomyocytes to shut down glycolysis and initiate lipid oxidation. Failure to downregulate HAND1 and thereby initiate lipid oxidation is fatal, emphasizing that oxidative metabolism is a prerequisite for sufficient energy production in the postnatal heart. Moreover, PPAR (peroxisome proliferator‐activated receptor) signaling plays an important role in activating lipid metabolism.^17^

The profound changes in the energy metabolism of cardiomyocytes are associated with alterations in mitochondria: the fetal‐type mitochondria undergo mitophagy and are replaced with mature adult‐type mitochondria in a Parkin‐dependent process to allow more efficient ATP production.^18^ Increased oxidative metabolism promotes reactive oxygen species production and thereby induces a DNA damage response, which is believed to contribute to cardiomyocyte cell cycle arrest.^19^ On a molecular level, endogenous mechanisms known to regulate cardiomyocyte proliferation include neuregulin‐ERBB2 (erb‐b2 receptor tyrosine kinase 2) signaling, the Hippo‐YAP (YY1 associated protein 1) pathway, and the transcription factor GATA4 (GATA binding protein 4), among others.^11^, ^20^, ^21^, ^22^ Moreover, even though metabolic pathways are controlled by the same signals that regulate cell proliferation,^23^ their significance in cardiac regeneration and cardiomyocyte proliferation is unknown.

In search of regenerative therapies, a number of studies have utilized transcriptomics to identify mechanisms regulating cardiac regeneration (for recent examples, see Natarajan et al,^24^ Quaife‐Ryan et al,^25^ and Kang et al^26^). However, proteomic and metabolomic changes have not been thoroughly investigated. In this article, we report the first integrated study combining genomewide RNA sequencing (RNAseq), global proteomics, and untargeted metabolomics to characterize in detail the metabolic changes occurring throughout the early postnatal heart development.

## Methods

The data, analytic methods, and study materials have been made available to other researchers for purposes of reproducing the results or replicating the procedure. Detailed methods are available in Data S1, and detailed protocols are available from the corresponding authors on reasonable request. The RNAseq data have been made publicly available at the National Center for Biotechnology Information gene expression and hybridization array data repository GEO.^27^ The proteomics raw data have been made available at the MassIVE repository.^28^ All other omics data are available in Appendices S1 through S5. The images and raw quantification data for cell viability and proliferation experiments are available from the corresponding authors on reasonable request.

**Figure 1:**
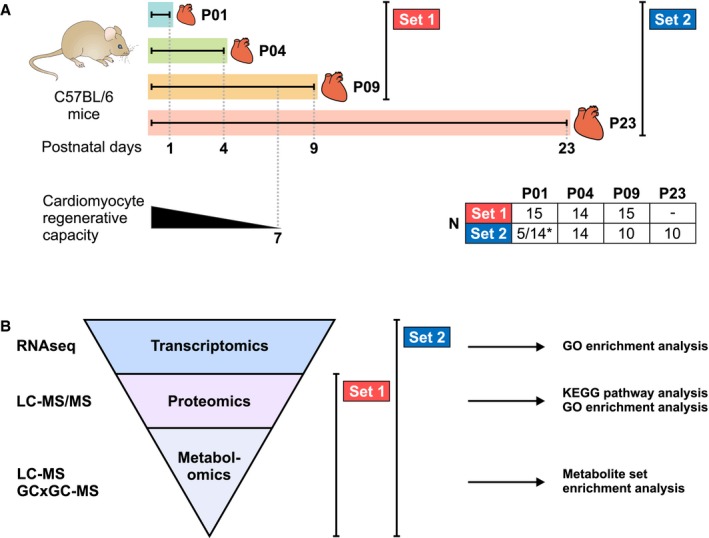
Experimental design for the multiomics analysis of postnatal mouse hearts. A, Two separate sets of mouse ventricular tissue samples collected on postnatal day 1 (P01), P04, P09, and P23 were used. The postnatal loss of cardiac regenerative capacity is illustrated for comparison, and numbers of animals in each sample group are presented in the table. *Total sample sizes are indicated for metabolomics (5) and proteomics (14). B, Analysis techniques and bioinformatics analyses used in the study. GCxGC‐MS, 2‐dimensional gas chromatography–mass spectrometry; GO, gene ontology; LC‐MS, liquid chromatography–mass spectrometry; LC‐MS/MS, liquid chromatography–tandem mass spectrometry; RNAseq, RNA sequencing.

The experimental design is summarized in Figure 1. Two separate sets of mouse (strain C57BL/6JOlaHsd, both sexes) ventricular tissue samples were used. Animal handling and all procedures were carried out in accordance with University of Helsinki institutional guidelines. Set 1 encompassed samples from postnatal day 1 (P01), P04, and P09, representing hearts with full regenerative capacity (P01), partial regenerative capacity (P04), and negligible regenerative capacity (P09), and was used for proteomics and metabolomics. To validate the results, a second set of samples was analyzed. In set 2, an additional sample group from 23‐day‐old mice (P23) was included to discriminate phenomena related to heart growth. The set 2 samples were subjected to transcriptomics using pooled samples (3 hearts per sample) and proteomics and metabolomics analyses (without pooling). The results from both sample sets are presented for proteomics and metabolomics, as the combination of both sample sets in metabolomics analyses using the linear mixed effects model produced more reliable results than either of the 2 sample sets alone. The methods and bioinformatics analyses are shown in Figure 1B. For transcriptomics analyses, statistically significant differential expression was defined as fold change >1.5 and q<0.01. For proteomics, metabolomics and bioinformatics analyses, q<0.01 with no fold change limit was considered statistically significant.

## Results

### Transcriptomics

**Figure 2:**
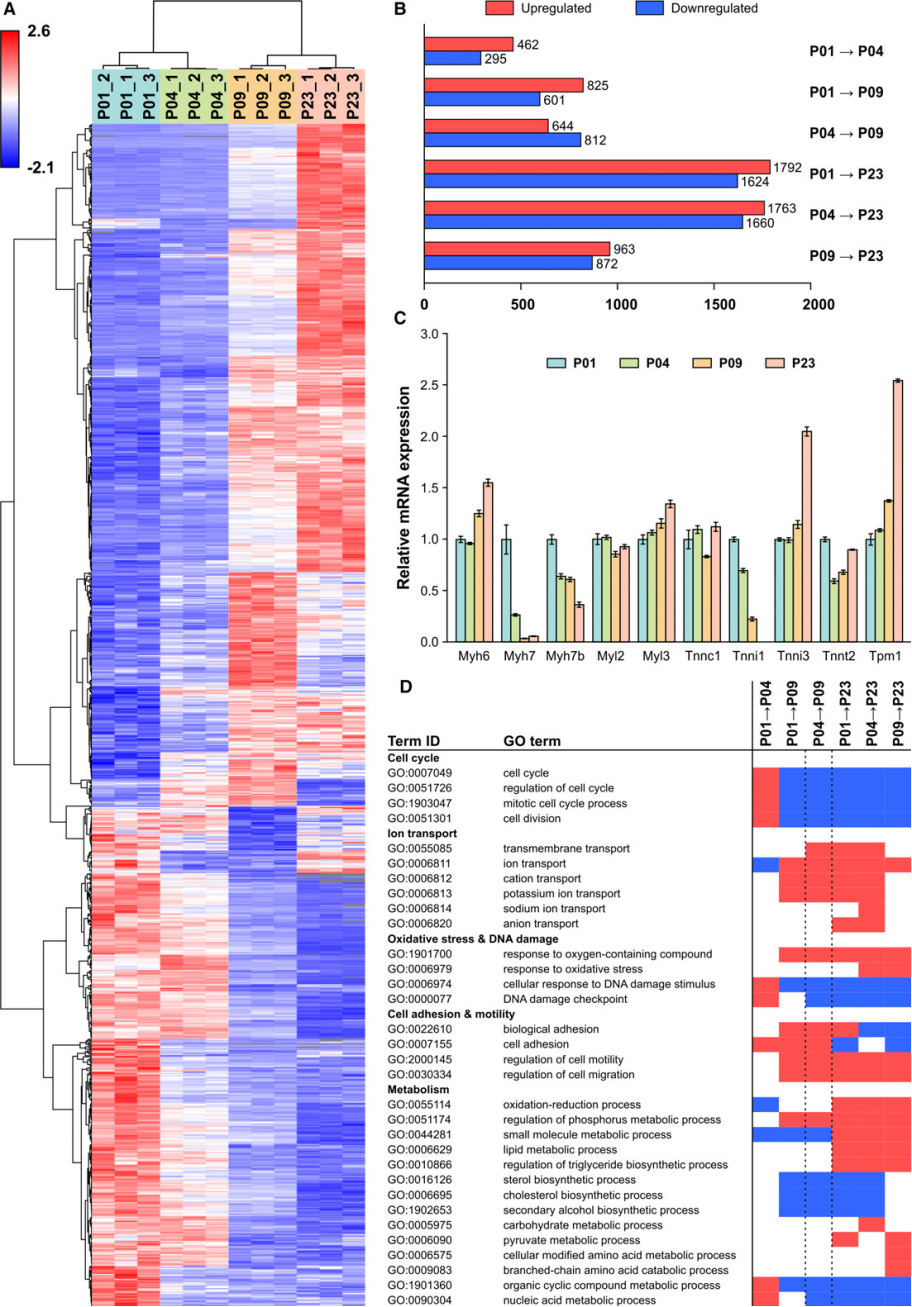
Gene expression changes in the neonatal mouse heart. A, Heat map of the top 1000 genes with the smallest q values between postnatal day 1 (P01) and P04. B, The numbers of up‐ and downregulated genes (q<0.01, fold change >1.5). C, Expression patterns of selected cardiomyocyte‐specific structural proteins. The data were normalized to P01 and are expressed as mean±SEM (n=3 pooled samples, each from 3 hearts). D, Selected significantly enriched (q<0.01) biological process gene ontology (GO) terms for each time point comparison from gene set enrichment analysis. Blue indicates downregulation, and red indicates upregulation. All gene symbol explanations are available in Appendix S1.

To analyze postnatal gene expression changes, RNAseq was carried out with ventricular tissue samples using 3 pooled samples from 9 hearts at each time point. Principal component analysis of RNAseq data showed clear grouping of samples and separation of sample groups (Figure S1). Figure 2A presents hierarchical clustering and a heat map of 1000 genes with most significant changes between P01 and P04. In total, 8547 individual protein‐coding genes were up‐ or downregulated statistically significantly (q<0.01, fold change >1.5); their numbers for each time‐point comparison are presented in Figure 2B. The greatest changes in gene expression were observed between time‐point comparisons P01 to P23 and P04 to P23. The top 10 up‐ and downregulated genes are presented in Figure S2.

The expression levels of cardiomyocyte‐specific structural proteins exhibited an anticipated pattern with a switch from *Myh7* to *Myh6* (myosin heavy chain 6 and 7, respectively) and from *Tnni1* to *Tnni3* (troponin I1 and troponin I3, respectively) within the early postnatal period (Figure 2C).^29^, ^30^ In addition, the expression profiles of the main cardiomyocyte ion channels (Figure S3A) were in line with previous reports.^31^ The expression patterns of control genes are presented in Figure S3B. Expression of *Actb* (actin beta), *Rpl4* (ribosomal protein L4), *Rpl32* (ribosomal protein L32), *Tbp* (TATA‐box binding protein), *Oaz1* (ornithine decarboxylase antizyme 1), and *Pgk1* (phosphoglycerate kinase 1) did not change significantly, whereas several other genes either generally used or recommended as control genes,^32^ such as *Gapdh* (glyceraldehyde‐3‐phosphate dehydrogenase), were up‐ or downregulated (q<0.01 and fold change >1.5) in at least 1 time‐point comparison. Furthermore, genes known to play a role in cardiomyocyte proliferation (*Erbb2*,* Gata4*), metabolic switch from glycolysis to fatty acid oxidation (*Hand1*,* Hif1a*,* Ppar* isoforms), and mitochondrial maturation (*Park2*, parkin RBR E3 ubiquitin protein ligase; *Pink1*, PTEN induced kinase 1) exhibited expected changes (Table S1). Differential expression analysis of all protein‐coding genes is available in Appendix S1.

To investigate biological processes linked to the observed gene expression changes, we carried out gene ontology (GO)^33^, ^34^ enrichment analysis, and changes with q<0.01 were considered significant. Changes in biological processes linked to cell proliferation, cardiac muscle, cell adhesion and motility, ion transport, and development of immune response were highlighted among the enriched GO terms (Figure 2D and Appendix S2). In accordance with increased oxidative metabolism, genes linked to oxidative stress were upregulated at P23 compared with P04 or P09. DNA damage response was upregulated from P01 to P04, whereafter it was downregulated in all other comparisons. Of the GO terms associated with cellular metabolism, oxidation‐reduction processes were upregulated at P23 compared with P01, P04, and P09; carbohydrate metabolism was upregulated from P04 to P23; and pyruvate metabolism was upregulated from P01 to P23 and P09 to P23. Sterol biosynthesis was downregulated in all other time‐point comparisons except P01 to P04 and P09 to P23, indicating that the transcript‐level changes take place mainly between P04 and P09. Amino acid metabolism and branched chain amino acid (BCAA) catabolism were upregulated from P09 to P23.

### Proteomics

**Figure 3:**
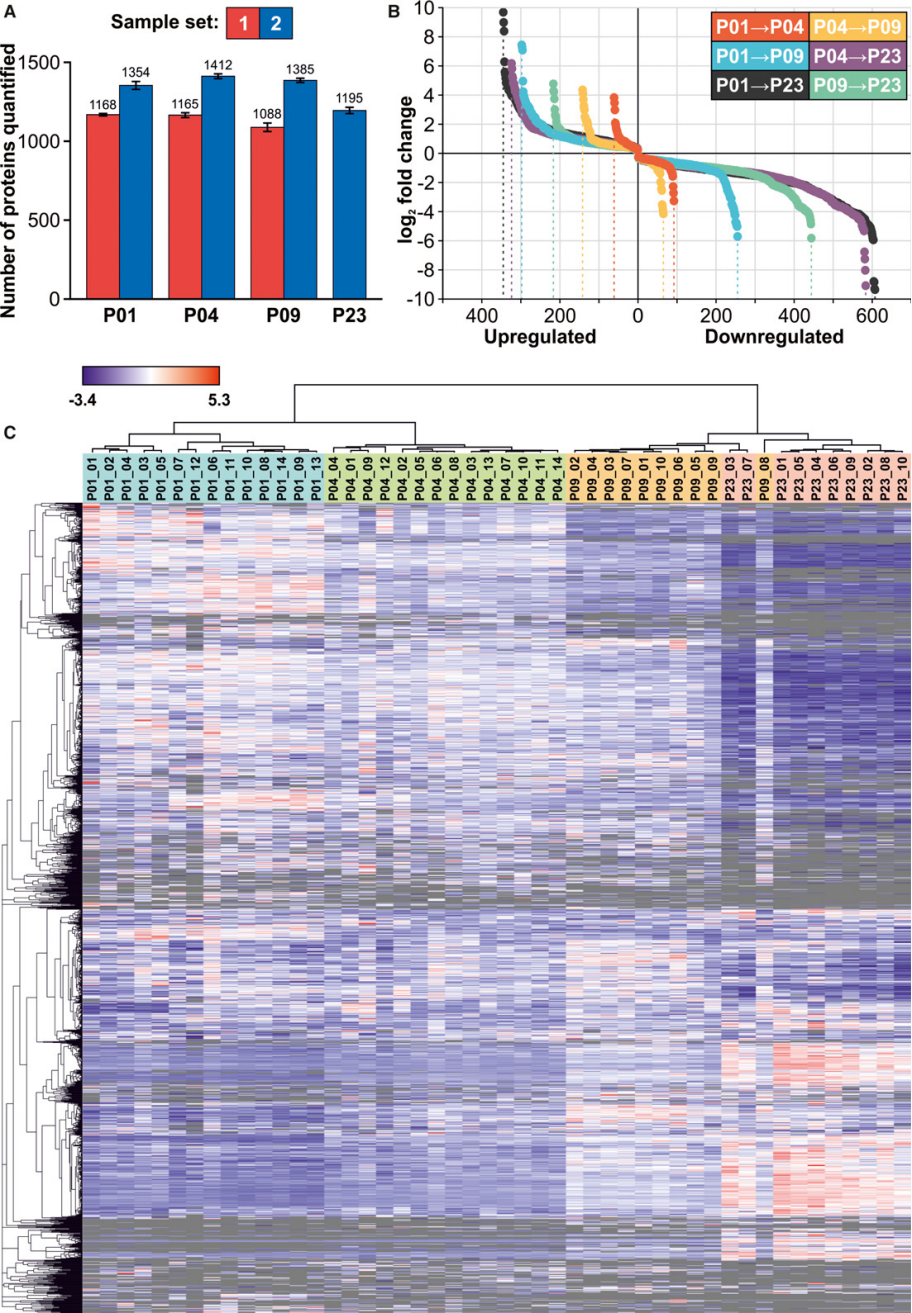
Proteomic changes in the neonatal mouse heart. A, The number of proteins quantified in each sample group, expressed as mean±SEM (identification false discovery rate <0.01 on both peptide and protein levels). B, The numbers and fold changes of differentially expressed (q<0.01) proteins in sample group comparisons. C, Hierarchical clustering of proteins and samples. All proteins detected in more than 67% of samples of at least 1 sample group are included in the heat map. Gray indicates missing values (protein not quantified). B and C, Data presented are from set 2; corresponding images for set 1 are in Figure S4. P indicates postnatal day.

To quantify protein abundances in ventricular tissue, we used a shotgun proteomics approach. On average, 1140 and 1337 distinct proteins were quantified in individual samples in sample sets 1 and 2, respectively (Figure 3A). The numbers and fold changes of differentially expressed proteins are shown in Figure S4A (set 1) and Figure 3B (set 2). The corresponding lists of differentially expressed proteins, as well as all quantified proteins and their label‐free quantification intensities, are presented in Appendix S3. Hierarchical clustering of the data shows that the sample groups were separated from each other in both sample sets (Figure 3C and Figure S4B); this was also seen in principal component analysis (Figure S5). GO enrichment analysis and KEGG^35^ pathway analysis were used to identify up‐ and downregulated processes and activated or inactivated pathways (Appendix S3). Oxidative metabolism and lipid metabolism were highlighted as upregulated phenomena at P04, P09, and P23 compared with P01, whereas in the downregulated GOs, nucleic acid metabolism and glycolysis were highlighted from P01 to P04, peptide metabolism and protein synthesis were highlighted from P01 to P09, and RNA processing and peptide metabolism were highlighted from P01 to P23.

The postnatal increased activity of energy metabolism and the shift from carbohydrates to fatty acids as the primary source of ATP were well represented in the proteomics data. Several components (ENO1 (enolase 1), PFKL (phosphofructokinase, liver type), PGK1 (phosphoglycerate kinase 1)) of the HIF1 signaling pathway that promotes anaerobic metabolism in the embryonic heart were downregulated after birth (Table S2). Furthermore, many key proteins related to fatty acid metabolism, degradation, and oxidative phosphorylation were upregulated after P01. This was paralleled with upregulation of most of the detected components of PPAR signaling, which promotes fatty acid oxidation, after P01. In line with the increasing mitochondrial content and maturation in cardiomyocytes, most mitochondrial proteins were highly upregulated from P01 to P23. These included not only enzymes linked to oxidative metabolism but also structural (IMMT (inner membrane mitochondrial protein)) and regulatory (PHB (prohibitin), DNAJA3 (DnaJ heat shock protein family (Hsp40) member A3)) proteins (Table S2). Furthermore, the mitochondrial isoforms of SOD2 (superoxide dismutase) and peroxiredoxins 3 and 5 (PRDX3, PRDX5) were strongly upregulated at P09 and P23 (Table S2), reflecting a response to oxidative stress.

Analysis of key proteins of cell cycle regulation and signaling pathway activation with proteomics is challenging because of the low abundance of these proteins. Nevertheless, expression of proteins related to microtubule dynamics during mitosis (DYNC1H1 (dynein cytoplasmic 1 heavy chain 1), PAFAH1B1 (platelet activating factor acetylhydrolase 1b regulatory subunit 1), RHOA (ras homolog family member A), and BUB3 (BUB3, mitotic checkpoint protein)) decreased with increasing postnatal age (Table S2). Most of the detected 14‐3‐3 proteins (YWHAB, YWHAE, YWHAH, YWHAQ, and YWHAZ (tyrosine 3‐monooxygenase/tryptophan 5‐monooxygenase activation proteins beta, epsilon, eta, theta and zeta)), some of which regulate cell cycle and cardiomyocyte proliferation,^36^, ^37^ were constantly downregulated from P01 to P23 in set 2 samples (Table S2). Components of the canonical Wnt pathway were also detected (Table S2), and the decreased abundance of β‐catenin (CTNNB1) and its nuclear interaction partner Pontin52 (RUVBL)^38^ indicate attenuation of canonical Wnt pathway activity from P01 to P09.

### Metabolomics

**Figure 4:**
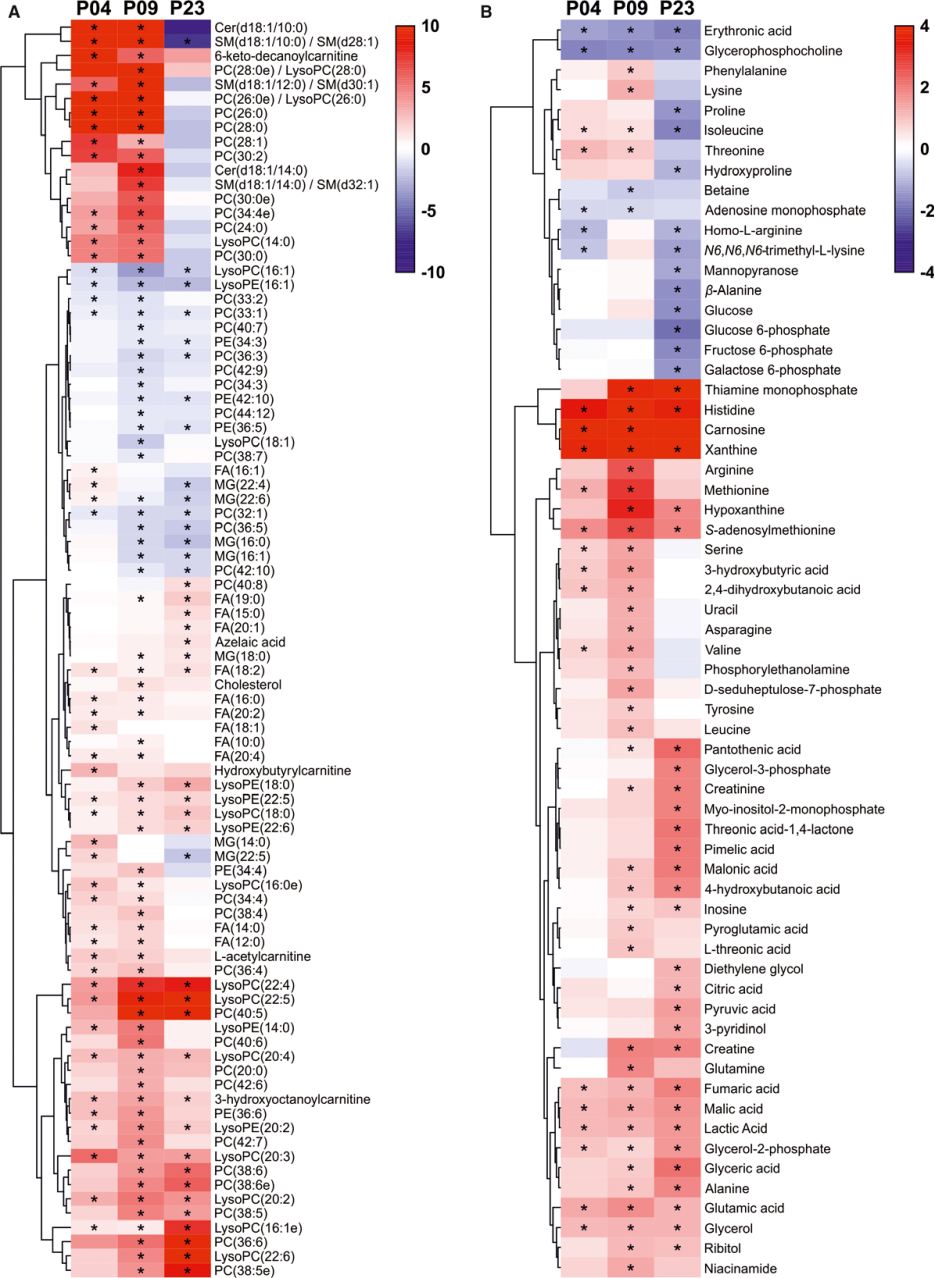
Metabolite changes in the postnatal mouse heart. A, Heat map of linear mixed effect (LME) model estimates for lipids. B, Heat map of LME model estimates for polar metabolites with Glass's Δ effect sizes from postnatal day 1 (P01). *q<0.01 compared with P01. Cer indicates ceramide; FA, fatty acid; LysoPC, lysophosphatidylcholine; LysoPE, lysophosphatidylethanolamine; MG, monoacylglycerol; PC, phosphatidylcholine; PE, phosphatidylethanolamine; SM, sphingomyelin.

For the untargeted metabolomics analysis, we used 2 complementary techniques to increase the metabolite coverage, namely, liquid chromatography‐mass spectrometry (LC‐MS) and 2‐dimensional gas chromatography coupled to MS (GC×GC‐MS), and combined the data from the 2 sample sets using the linear mixed effects model. Quality control results are available in Data S1. In total, 805 and 162 metabolic features changed statistically significantly (q<0.01) in at least 1 of the group comparisons (P01→P04, P01→P09, P01→P23) for analyses with LC‐MS and GC×GC‐MS, respectively (Appendix S4). Based on the MS/MS analysis of metabolic features, 151 significantly up‐ or downregulated metabolites were identified and are shown in Figure 4.

In line with the transcriptomics and proteomics results, the metabolomics data show a distinct transition from carbohydrates to lipids as the main source of energy. The abundances of glucose and sugar derivatives (glucose, glucose‐6‐phosphate, fructose‐6‐phosphate, galactose‐6‐phosphate) were downregulated at P23 compared with P01 (Figure 4B), whereas the abundances of most fatty acids and components of glycerolipid metabolism (glycerol‐3‐phosphate, glycerol‐2‐phosphate, glycerol, glyceric acid) increased after P01 (Figure 4A and 4B). The increased fatty acid β‐oxidation was also reflected as increased abundance of acylcarnitines at P04 and P09 (Figure 4A). However, most metabolites of the citric acid cycle (citric acid, fumaric acid, pyruvic acid, malic acid, lactic acid), pentose‐phosphate pathway (D‐seduloheptulose‐7‐phosphate), and glycolysis (lactic acid, pyruvic acid) exhibited a constant rise with increasing postnatal age (Figure 4B), reflecting a total increase in energy metabolism. No changes were detected in the levels of citric acid cycle metabolites succinic acid and α‐ketoglutarate (Appendix S4). These data indicate a transition from carbohydrates to fatty acids as the main source of energy and a total increase in energy metabolism over the early postnatal period.

In addition to the general increase in fatty acid abundance, various lipid species (phospholipids, lysophospholipids, monoacylglycerides, and fatty acids) displayed interesting changes in abundance (Figure 4A). For phospholipids, there was a general trend toward increased saturation level along with increasing postnatal age. All phospholipids showing a constant decrease from P01 to P23 were unsaturated, and many of them were polyunsaturated. The phospholipid species with saturated medium‐to‐long‐chain fatty acids exhibited an increase at P04 and P09 followed by a decrease at P23. Several lysolipid species (LysoPC and LysoPE) increased at P04 and remained constant or increased further through P09 to P23, with the exception of lysolipids with fatty acids 16:1 and 18:1, which decreased after P01, or 14:0, which decreased after P04. Interestingly, myristic acid (fatty acid 14:0) exhibited a comparable pattern both as a free fatty acid and when incorporated into other lipid species (eg, ceramide, sphingomyelin, or phosphocholine): The abundance increased at P04 and P09 and decreased at P23. A similar pattern was also observed for other medium‐chain saturated fatty acid species (Figure 4A).

The levels of most amino acids displayed an initial increase at P04 and/or P09 followed by a decrease at P23 (Figure 4B). In contrast to other (proteinogenic) amino acids, the abundance of glutamic acid, alanine and histidine increased initially but remained significantly higher at P23 compared with P01 (Figure 4B). The increase in amino acid abundance from P01 to P09 likely reflects the active protein synthesis required during cardiomyocyte growth and maturation.

We also observed significant changes in purine metabolism, particularly among the metabolites of AMP catabolism. The levels of AMP had decreased already at P04, paralleled by an increase in the abundance of its degradation pathway metabolites inosine, hypoxanthine, and xanthine (Figure 4B). This likely reflects the alterations in the energy metabolism, as high AMP abundance immediately after birth would promote fatty acid β‐oxidation through AMPK (AMP‐activated protein kinase).^39^ In line with the postnatal increase in cardiac workload, we also observed increased abundance of creatine and creatinine at P09 and P23 compared with P01 (Figure 4B). Other interesting metabolite findings include the increase of the ascorbic acid metabolites L‐threonic acid and threonic acid 1,4‐lactone on P09 and P23, respectively, reflecting increased oxidative stress with increasing postnatal age.

### Multiomics Integration

**Figure 5:**
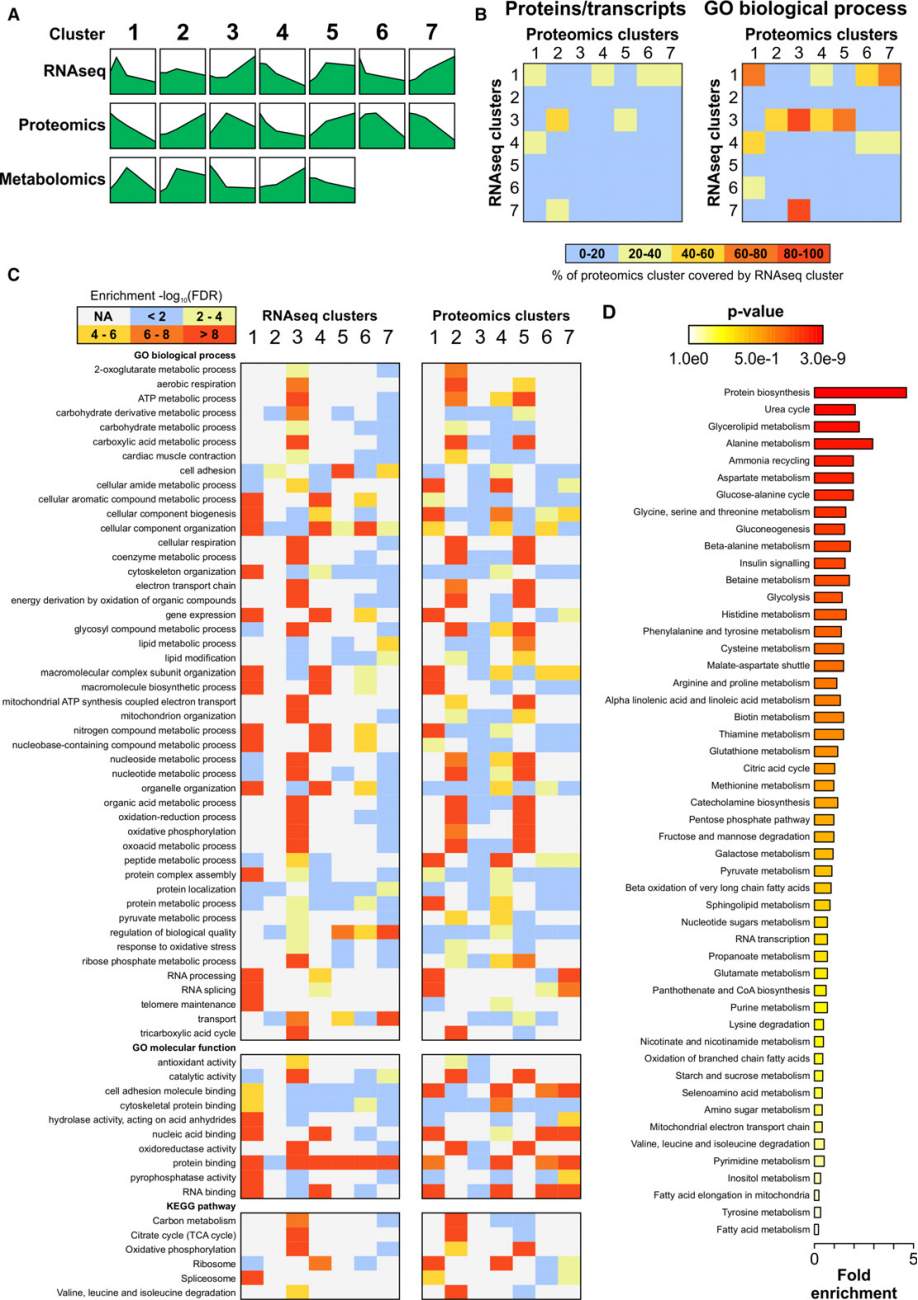
Multiomics integration with fuzzy c‐means clustering. A, Median abundance patterns of mRNAs, proteins, and metabolites in each cluster. Detailed images of clusters and the scales for the *y*‐axis are in Figure S6. B, The percentage of proteomics clusters covered by the RNA sequencing (RNAseq) clusters at the level of transcripts/proteins (left) and enriched biological process gene ontology (GO) terms (q<0.01) in the clusters (right). C, A heat map of −log_10_ false discovery rate (FDR) values of selected GO terms and KEGG pathways in RNAseq and proteomics clusters. D, Metabolite set enrichment analysis performed on the union of metabolites in all clusters indicating the biological processes associated with metabolite changes. CoA indicates coenzyme A; NA, not assessed.

For comprehensive multiomics integration, we utilized fuzzy c‐means clustering, in which the transcripts, proteins, and metabolites were assigned into ≥1 clusters based on their abundance patterns (Figure 5A, Figure S6). We compared transcriptomics and proteomics clusters on transcript/protein levels but also for enriched GO terms and KEGG pathways (q<0.01). The percentage of proteomics clusters covered by the RNAseq clusters are shown in Figure 5B for the proteins/transcripts and biological process GO terms and in Figure S7A for cellular component and molecular function GO terms and KEGG pathways. Selected enriched GO terms and KEGG pathways and their enrichment in each transcriptomics and proteomics cluster are presented in Figure 5C (cellular component GO terms in Figure S7B).

Because fuzzy clustering could only be carried out separately for the 2 sample sets, it provided only a little more insight to the metabolomics data compared with the linear mixed effects model. Consequently, instead of analyzing the individual metabolomics clusters, we performed metabolite set enrichment analysis on all significantly changed and identified metabolites to investigate the metabolic pathways with which the significantly up‐ or downregulated metabolites were associated. Based on metabolite set enrichment analysis, the 3 most significantly enriched metabolic pathways were protein biosynthesis, urea cycle, and glycerolipid metabolism (Figure 5D). Enrichment of multiple individual amino acid metabolic pathways was also observed. The high enrichment of urea cycle metabolites correlated with the changes in individual metabolites, such as arginine and fumarate (Figure 4B). Not all identified urea cycle metabolites, however, exhibited statistically significant changes (eg, aspartate and urea; Appendix S4).

Multiomics integration for the KEGG pathway “glycolysis and gluconeogenesis” is presented in Figure S8 as an example. In line with the data from the individual omics analyses, glycolysis was not uniformly activated or inactivated. Instead, the genes and proteins in the beginning and the end of the pathway were either upregulated at later time points or exhibited variable abundance patterns, whereas the genes and proteins in the middle of the pathway were either downregulated with increasing postnatal age or displayed variable expression patterns. Most enzymes of the related pyruvate metabolism pathway were also differentially expressed on mRNA and/or protein levels within the early postnatal period (Figure S9).

To identify potential upstream regulators of gene and protein expression changes in each RNAseq and proteomics cluster, we subjected the individual clusters to Ingenuity pathway analysis (Qiagen).^40^ Upstream regulators belonging to the classes *transcription regulator, microRNA*, and *chemical—endogenous mammalian* were included in the analyses. Of transcriptional regulators, HAND1 was identified as a potential upstream regulator for RNAseq cluster 5 and proteomics cluster 3 (Appendix S5), which is in line with its known role in the regulation of postnatal energy metabolism. In the microRNA analyses, miR‐1, miR‐21, miR‐122, and let‐7 were identified as potential upstream regulators of various mRNA and protein clusters (Appendix S5). Furthermore, the analysis of endogenous chemicals identified several interesting metabolites as potential regulators of up‐ and downregulated mRNAs and proteins—palmitic acid, cholesterol, fatty acid, amino acids, and butyric acid (Appendix S5)—correlating well with the metabolomics data. This multiomics integration and upstream regulator analysis prompted us to investigate postnatal changes in cardiac amino acid metabolism, fatty acid synthesis, mevalonate pathway (cholesterol synthesis), and ketogenesis in more detail.

### BCAA Catabolism

**Figure 6:**
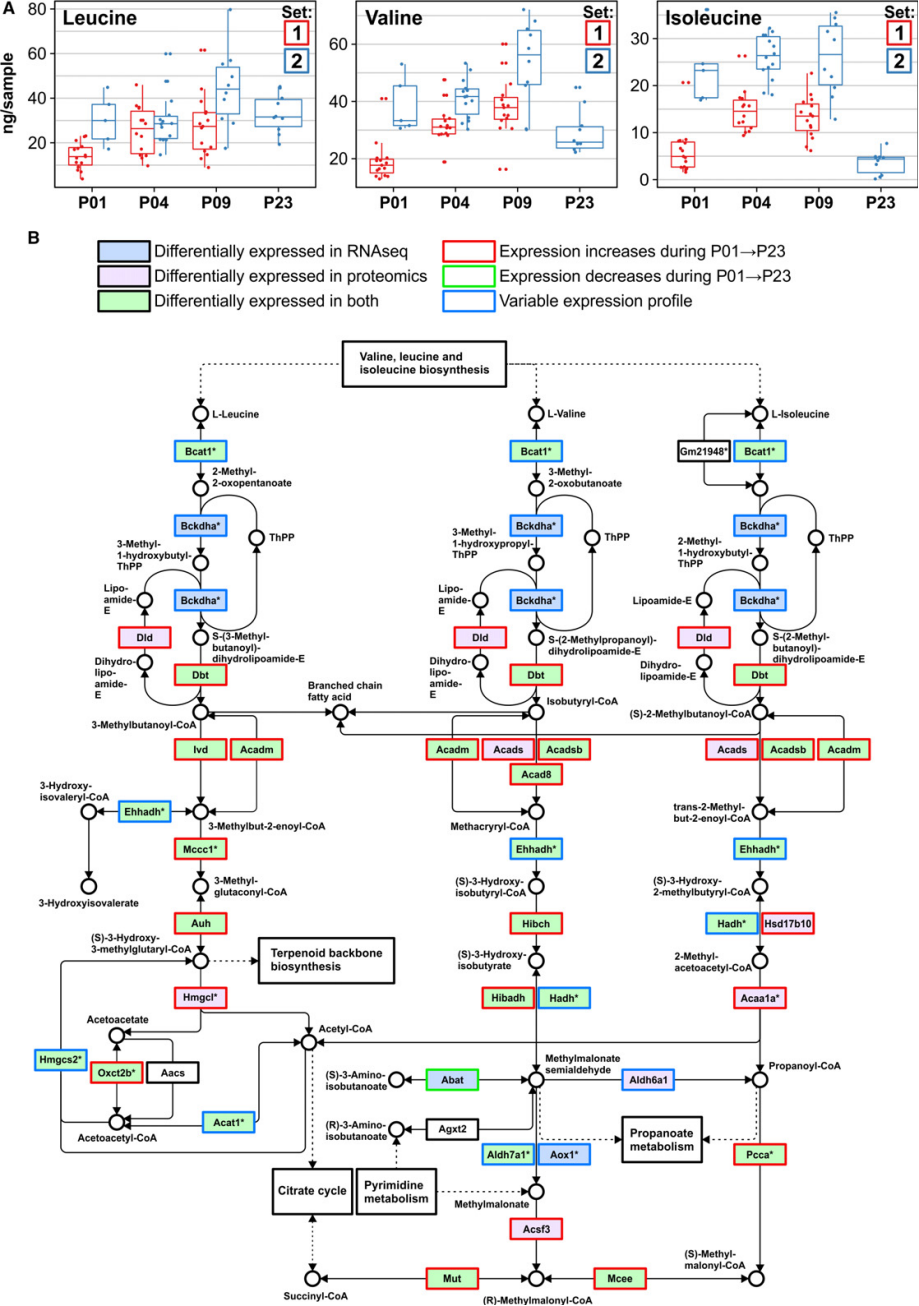
Postnatal changes in branched chain amino acid degradation in the mouse heart. A, Concentrations of branched chain amino acids valine, leucine, and isoleucine in mouse ventricular tissue. B, The KEGG pathway map of valine, leucine, and isoleucine degradation indicating up‐ and downregulated transcripts and proteins. *Multiple enzymes may catalyze the same reaction. All gene symbol explanations are available in Appendix S1. The KEGG pathway image is modified and reprinted with permission from the Kyoto Encyclopedia of Genes and Genomes.^65^ P indicates postnatal day; RNAseq, RNA sequencing.

To gain deeper insight into the significant enrichment of the KEGG pathway “valine, leucine, and isoleucine degradation” in transcriptomics and proteomics clusters showing upregulation throughout the postnatal period, we focused on BCAA catabolism in more detail. The concentrations of valine, leucine, and isoleucine increased from P01 to P09, after which they decreased to P01 levels or lower at P23 (Figure 6A). Most enzymes in the BCAA degradation pathway were significantly upregulated at P23 compared with P01 on either transcript or protein levels, or both (Figure 6B). On the mRNA level, most differentially expressed genes were upregulated between P09 and P23, thus correlating directly with the BCAA concentrations. Of the 37 quantified individual proteins on this pathway, 19 and 31 proteins were upregulated at P09 and P23, respectively, compared with P01 (set 2 samples; Appendix S3). The rate‐limiting step of BCAA catabolism is mediated by the branched‐chain α‐ketoacid dehydrogenase (BCKDC or BCKDH) complex, the activity of which is controlled by inactivating phosphorylation and activating dephosphorylation. Both the α subunit of the complex, *Bckdha*, and the protein phosphatase responsible for BCKDH activation (*Ppm1k*, protein phosphatase, Mg2+/Mn2+ dependent 1K) were significantly upregulated on the mRNA level at P23, and BCKDHA protein abundance was upregulated at P23 (PPM1K was not detected; Table S2).

### Fatty Acid Metabolism

**Figure 7:**
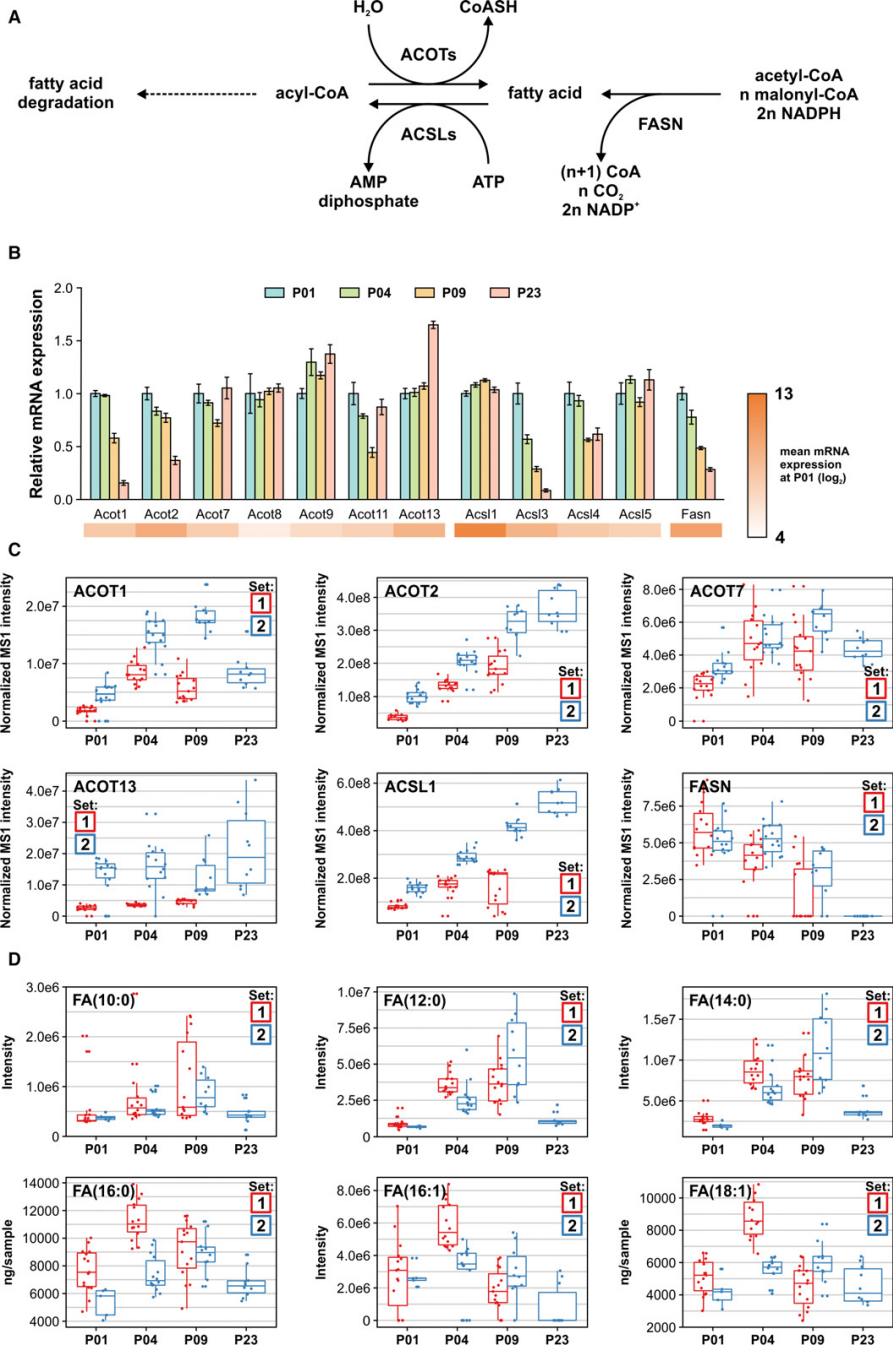
Postnatal changes in fatty acid metabolism in the mouse heart. A, The levels of free fatty acids (FAs) are regulated by ACOTs (acyl‐CoA [acyl‐coenzyme A] thioesterases) that hydrolyze acyl‐CoAs to CoASH (free coenzyme A) and free FAs, ACSLs (long‐chain FA–CoA ligases) that activate free FAs by ligation of CoA, and FASN (FA synthase). B, Relative mRNA expression of enzymes regulating the concentrations of free FAs, shown as mean±SEM (n=3 pooled samples, each from 3 hearts). C, Normalized label‐free quantification intensities of FA‐regulating enzymes detected in proteomics. D, The abundances or concentrations of selected free FAs. MS1 indicates precursor ion mass spectrum; P, postnatal day.

The main enzymes regulating the abundance of free fatty acids are FASN (fatty acid synthase); ACSLs (long‐chain acyl‐CoA [acyl‐coenzyme A] synthetases), which attach CoA to free fatty acids; and ACOTs (acyl‐CoA thioesterases), which remove the CoA from acyl‐CoA, thus releasing free fatty acid (Figure 7A). The relative mRNA levels of FASN, ACOTs, and ACSLs are presented in Figure 7B. Downregulation of *Acot1*,* Acot2*,* Acsl3, Acsl4*, and *Fasn* was observed after P01, whereas *Acot13* was upregulated at P23. In general, there was relatively poor correlation between protein (Figure 7C) and mRNA expression. The abundance of ACOT isoforms increased, except for ACOT1, which was first upregulated at P04 and P09, followed by downregulation at P23. The abundance of ACSL1 increased over the postnatal period. FASN, however, was strongly downregulated at P23 to undetectable levels, correlating with the mRNA expression pattern. The products of the cytosolic ACOT1 and mitochondrial ACOT2, saturated and monounsaturated medium‐to‐long‐chain fatty acids,^41^ exhibited almost identical patterns with an initial increase peaking at P04 or P09, followed by strongly diminished levels at P23 (Figure 7D) and thus correlating with ACOT1 abundance. Furthermore, most enzymes in the KEGG pathway “fatty acid degradation” were differentially expressed on mRNA and/or protein levels (Figure S10). These data reflect the complex regulation of fatty acid levels in the postnatal heart in response to the increased fatty acid abundance from nutrients.

### Mevalonate Pathway and Ketogenesis

**Figure 8:**
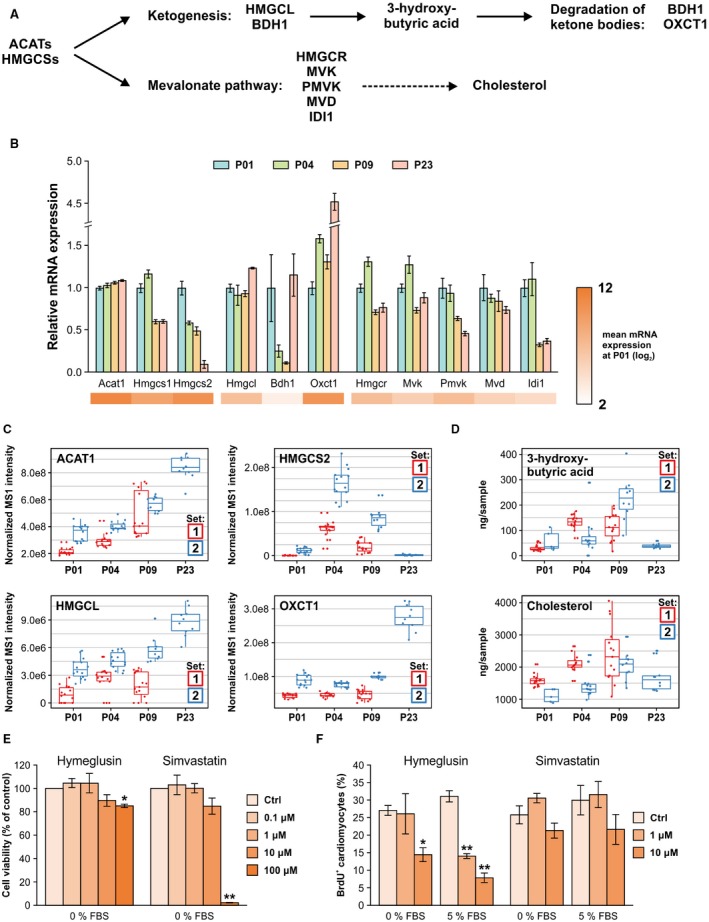
Ketogenesis and mevalonate pathways in the early postnatal heart. A, ACATs (acetyl‐CoA [acetyl‐coenzyme A] acetyltransferases) and HMGCSs (hydroxymethylglutaryl‐CoA synthases) catalyze HMG‐CoA (hydroxymethylglutaryl‐CoA) synthesis. HMG‐CoA serves as a substrate for the ketogenesis route producing 3‐hydroxybutyrate and the mevalonate pathway producing mevalonate, which can be further used for cholesterol synthesis. B, Relative mRNA expression of selected ketogenesis and mevalonate pathway components, shown as mean±SEM (n=3 pooled samples, each from 3 hearts). C, Normalized label‐free quantification intensities of proteins detected in the ketogenesis and mevalonate pathways. D, Concentrations of 3‐hydroxybutyrate and cholesterol over the early postnatal period. E and F, Effect of HMGCS inhibition with hymeglusin and HMGCR (HMG‐CoA reductase) inhibition with simvastatin on neonatal rat ventricular cardiomyocyte viability (E) and proliferation (F). Cell viability was assessed using the MTT assay, and cell proliferation was quantified as the percentage of BrdU‐positive cells after 24‐hour exposure. The data are expressed as mean±SEM from 3 independent experiments. *P<0.05, **P<0.01 compared with control (Ctrl); Welch ANOVA followed by Games‐Howell. All gene symbol explanations are available in Appendix S1. BDH1 indicates 3‐hydroxybutyrate dehydrogenase 1; FBS, fetal bovine serum; HMGCL, hydroxymethylglutaryl–coenzyme A lyase; IDI1, isopentenyl‐diphosphate δ‐isomerase 1; MVD, mevalonate diphosphate decarboxylase; MS1, precursor ion mass spectrum; MVK, mevalonate kinase; OXCT1, 3‐oxoacid CoA‐transferase 1; PMVK, phosphomevalonate kinase.

Because HMGCS2 (HMG‐CoA [hydroxymethylglutaryl‐CoA] synthase 2) was the most significantly upregulated protein from P01 to P04 and cholesterol biosynthesis was one of the enriched metabolism‐related biological processes in GO enrichment analysis, the HMGCS‐mediated mevalonate pathway and ketogenesis were investigated in more detail. The HMGCS‐catalyzed synthesis of HMG‐CoA serves as a substrate for HMGCR (HMG‐CoA reductase) in the mevalonate pathway and HMGCL (HMG‐CoA lyase) in ketogenesis (Figure 8A). Both the cytosolic *Hmgcs1* and the mitochondrial *Hmgcs2* isoforms were downregulated at the mRNA level at P09 and P23 compared with P01, whereas there was no change in the mRNA levels of the upstream enzyme ACAT1 (acetyl‐CoA acetyltransferase; Figure 8B). Of the enzymes regulating ketone body abundance, expression of *Bdh1* was significantly lower at P09 compared with P01 or P23, and the expression of *Oxct1*, which oxidizes ketone bodies, was upregulated after P09. In the mevalonate pathway, *Hmgcr* expression was downregulated at P09 compared with P01 or P04 and at P23 compared with P04. Similarly, *Mvk* (mevalonate kinase), *Pmvk* (phosphomevalonate kinase), and *Idi1* (isopentenyl‐diphosphate δ‐isomerase 1) were downregulated with increasing postnatal age. Of these enzymes, ACAT1, HMCSS2, HMGCL, and OXCT1 (3‐oxoacid CoA‐transferase 1) were reliably quantified in proteomics (Figure 8C). The abundances of ACAT1 and HMGCL increased throughout the early postnatal period, whereas HMGCS2 peaked at P04 and was downregulated to undetectable levels by P23 and OXCT1 was upregulated from P09 to P23. At the metabolite level, the end product of ketogenesis, 3‐hydroxybutyric acid (β‐hydroxybutyrate), peaked at P09 and was downregulated at P23 (Figure 8D), correlating with changes in the abundance of HMGCS2, which is the rate‐limiting enzyme of ketogenesis, and OXCT1. Metabolites of the mevalonate pathway were not detected or identified; however, the concentrations of cholesterol, which is produced from mevalonate, increased at P09 and decreased thereafter (Figure 8D). Collectively, these results show that the HMGCS‐mediated mevalonate pathway and ketogenesis are activated transiently after birth in the mouse heart.

To evaluate the role of mevalonate pathway and ketogenesis in cardiomyocyte proliferation, we investigated the effects of pharmacological HMGCS and HMGCR inhibition on the viability and proliferation of neonatal rat ventricular cardiomyocytes. The HMGCS inhibitor hymeglusin^42^ induced a ≈15% decrease in cell viability at 100 μmol/L but had no effect at lower concentrations (Figure 8E and 8F). However, simultaneous inhibition of the mevalonate and ketogenesis routes with hymeglusin decreased the percentage of BrdU‐positive cardiomyocytes in both serum‐free and serum‐stimulated conditions already at smaller, nontoxic concentrations (10 and 1–10 μmol/L, respectively; Figure 8E and 8F). Inhibition of the mevalonate pathway alone using the HMGCR inhibitor simvastatin, however, had no effect on the percentage of BrdU‐positive cardiomyocytes at nontoxic concentrations (Figure 8E). These results indicate that the HMGCS‐mediated ketogenesis may participate in regulating cardiomyocyte cell cycle activity.

## Discussion

Therapeutic strategies to promote regeneration of the adult human hearts are urgently sought.^12^, ^13^ Nevertheless, more detailed understanding of the metabolic changes and signaling pathways mediating cardiomyocyte maturation and cell cycle withdrawal is required for the development of regenerative therapies. In this work, we employed an integrated multiomics approach to investigate the metabolic changes occurring in the mouse heart within the early postnatal period to identify metabolic pathways associated with the postnatal loss of regenerative capacity. This study provides an important resource for molecule (RNA, protein, and metabolite) abundances in the neonatal mouse heart. Furthermore, we highlighted examples of metabolic pathways that exhibited correlative changes on all 3 levels.

According to previous reports and the present data, the cardiac energy metabolism changes drastically after birth in response to altered nutrient availability and transition to oxygen‐rich environment. Increased oxidative metabolism gives rise to reactive oxygen species causing oxidative stress and DNA damage, which is thought to contribute to cardiomyocyte cell cycle withdrawal.^19^, ^43^ Remarkably, exposure of adult mice to chronic hypoxia reduces oxidative metabolism and DNA damage and promotes cardiac regeneration after MI.^44^ Oxidative DNA damage does not, however, correlate directly with cardiomyocyte cell cycle withdrawal in humans.^45^ The fact that human pluripotent stem cell–derived cardiomyocytes continue to respond to mitogenic stimuli despite a normoxic environment may also indicate that other mechanisms contribute to the irreversible cell cycle withdrawal. Nevertheless, the metabolic switch from glycolysis to fatty acid oxidation, achieved by increased palmitic acid availability and insulin depletion, was recently reported to be sufficient for inducing irreversible cell cycle exit of human pluripotent stem cell–derived cardiomyocytes, indicating that oxidative metabolism plays a major role in regulating cardiomyocyte cell cycle.^46^ Furthermore, increased fatty acid abundance has been shown to mediate postprandial physiological cardiac hypertrophy in the Burmese python.^47^ Administration of a combination of myristic, palmitic, and palmitoleic acid to mice or pythons also induces cardiac hypertrophy without pathological fibrosis or activation of the fetal gene program. In this study, we showed a temporally regulated postnatal increase in the abundance of saturated and monounsaturated medium‐chained fatty acids—both as free fatty acids and in various lipid species such as ceramides, sphingomyelins, and phosphocholines. This transient increase could serve as the physiological mechanism regulating nonpathological cardiomyocyte hypertrophy during postnatal heart growth, as reported for the Burmese python.^47^ It is also tempting to speculate that the same fatty acids may play a role in driving postnatal cardiomyocyte cell cycle withdrawal, as reported for human pluripotent stem cell–derived cardiomyocytes.^46^

Another key finding of this work, not previously described in the context of postnatal heart development, is the temporal regulation of the mevalonate pathway, which was strongly activated immediately after birth and downregulated after the regenerative window. The mevalonate pathway plays a role in cancer cell proliferation and is upregulated by several oncogenic signaling routes.^48^ Furthermore, its downregulation has been linked to increased cell size in vivo,^49^ and inhibition of HMGCR, the rate‐limiting enzyme of mevalonate pathway, attenuates cell proliferation and increases cell size in various cell types in vitro.^50^ Under our experimental conditions, specific inhibition of the mevalonate pathway with simvastatin, however, had no effect on neonatal cardiomyocyte proliferation, indicating that the active mevalonate pathway is dispensable for cardiomyocyte proliferation.

Parallel to the mevalonate pathway, we observed transient postnatal activation of ketogenesis in the mouse heart. The circulating levels of β‐hydroxybutyrate, which is mainly produced in the liver, increase temporarily after birth and provide an important source of energy for the developing brain.^51^ However, the observed strong temporal upregulation of HMGCS2, the rate‐limiting enzyme of ketogenesis, indicates that ketone body synthesis is also locally regulated and postnatally activated in the myocardium. In addition to its role as a circulating energy source, β‐hydroxybutyrate participates in cellular signaling by acting on cell membrane receptors and by directly inhibiting histone deacetylases,^52^ which has been reported to suppress oxidative stress.^53^ Increased abundance of ketogenic enzymes has been linked to aggressiveness of prostate cancer,^54^ indicating that augmented ketogenesis may provide a proliferative advantage in certain conditions. We further showed that simultaneous inhibition of the mevalonate pathway and ketogenesis attenuates neonatal cardiomyocyte proliferation in vitro. Because inhibition of the mevalonate route with simvastatin had no effect on cardiomyocyte proliferation, our results suggest that the observed transient postnatal upregulation of ketogenesis in the postnatal mouse heart may participate in regulating the cardiomyocyte cell cycle. It is tempting to speculate that by inhibiting HMGCS, hymeglusin could reduce the production of β‐hydroxybutyrate, which is known to suppress oxidative stress through inhibition of histone deacetylases, and thereby increase oxidative stress and DNA damage and promote cardiomyocyte cell cycle withdrawal. Clarification of the exact mechanisms requires further investigation.

Unlike other amino acids, the BCAAs valine, leucine, and isoleucine are metabolized mainly in organs other than the liver, such as skeletal and cardiac muscle. They provide nitrogen for maintaining glutamate, alanine, and glutamine pools and function as signaling molecules activating mTOR (mammalian target of rapamycin) signaling, which regulates cardiac homeostasis and plays a crucial role in cardiac pathophysiology.^55^, ^56^ In addition, isoleucine has also been reported to inhibit the transport and utilization of fatty acids in skeletal muscle in mice.^57^ The observed increase in BCAA degradation in the postnatal heart is thus in line with the decreased protein synthesis at P23 and the increase in fatty acid metabolism after P01. Even though the abundance of BCAAs is altered in experimental models of pressure overload and MI,^58^ the postnatal changes observed in the present study are unlikely to contribute to the postnatal loss of cardiac regeneration, as they predominantly take place after the regenerative window.

With respect to methodological limitations, proteomic and metabolomic analyses can detect only a fraction of proteins and metabolites. By using shotgun proteomics, we were able to perform relative quantification of >2000 proteins. Hydrophobic (eg transmembrane) proteins and proteins with very low abundance, for example, were often below detection. To increase metabolite coverage, we applied 2 complementary MS‐based methods for the metabolomics analyses. Metabolite identification was confined to previously reported metabolites in spectral libraries; therefore, numerous detected metabolic features with interesting abundance patterns remain unidentified. In general, the correlation between mRNA and protein levels is only modest, which can be caused by, for example, variable protein turnover rates. Integration of protein turnover analysis to transcriptomics and proteomics was recently shown to increase the yield of identified disease gene candidates in pathological cardiac hypertrophy in mice,^59^ highlighting the benefit of adding further layers to omics‐based analyses. Qualitative and quantitative assessment of all biological processes has been deemed essential for the investigation of cardiac metabolism by the American Heart Association.^60^ In the present study, integration of 3 omics analyses with 4 time points over the early postnatal period provides a comprehensive view to the metabolic changes taking place in the developing heart. Interestingly, in some cases we observed surprisingly long delays in the abundance changes from mRNA to protein and from protein to metabolite, as exemplified by the mevalonate pathway and ketogenesis. This highlights how crucial it is not only to use several omics methods but also to include several time points when analyzing dynamic phenomena with integrative systems biology approaches.

Considering the multicellular composition of the heart, the present study cannot elucidate cardiomyocyte‐specific phenomena because the data represent tissue‐level changes. In cell volume, cardiomyocytes build up 70% to 80% of the cardiac tissue,^61^ whereas in cell numbers, cardiomyocytes constitute roughly one third of the cell population, and endothelial cells represent the most abundant cell type, with a ≈45% proportion.^62^ These 2 cell types play a crucial role in postinfarction cardiac regeneration and, unlike fibroblasts or leukocytes, fail to initiate the neonatal‐type gene expression response upon injury in adult hearts.^25^ In cardiomyocytes, this was suggested to result from chromatin inaccessibility and thus to represent an epigenetic roadblock that prevents cardiomyocyte cell cycle reentry. Recent evidence suggests that metabolic pathways are intimately connected with epigenetic regulation.^63^, ^64^ Whether the postnatal metabolic remodeling is in fact the driving force that induces cardiomyocyte cell cycle exit requires further investigation.

In summary, we used an integrative systems biology approach with 3 levels of omics analyses to characterize changes in molecule abundances throughout the early postnatal period and to identify metabolic pathways associated with cardiac regeneration. To our knowledge, this study is the first combining transcriptomics with untargeted proteomics and global metabolomics analyses over several time points in the early postnatal heart and, as such, provides an extensive resource for molecule abundances for future mechanistic studies. Furthermore, we present several examples of metabolic pathways with correlative changes on all omics levels. These include the well‐established metabolic switch from glycolysis to fatty acid β‐oxidation as well as many previously unreported changes in cardiac metabolic pathways, such as the mevalonate pathway and ketogenesis. Finally, we identified a biological function for mevalonate and ketone body metabolism in the heart with a potential role in the regulation of neonatal cardiomyocyte proliferation. This integrated molecule‐level data may open up new possibilities for the development of regenerative therapies.

## Sources of Funding

This study was supported by Business Finland (Tekes; project no. 40395/13, 3iRegeneration); the Finnish Foundation for Cardiovascular Research; the Sigrid Jusélius Foundation; and the Academy of Finland (project no. 2666621).

## Disclosures

None.

## Supporting information


**Data S1.** Supplemental methods.
**Table S1.** Expression of Selected Genes Linked to Cardiac Regeneration and the Postnatal Metabolic Switch
**Table S2.** Proteomics
**Figure S1.** Individual factor maps of the RNA sequencing data principal component (PC) analysis shows perfect separation of sample groups.
**Figure S2.** Top 10 down‐ and upregulated genes in the postnatal mouse heart.
**Figure S3.** Ion channel and control gene expression in the postnatal mouse ventricular tissue.
**Figure S4.** Proteomic changes in neonatal mouse hearts.
**Figure S5.** Individual factor maps of the proteomics data principal component (PC) analysis of sample set 1 (A) and sample set 2 (B).
**Figure S6.** The abundance profiles of all individual transcripts, proteins, and metabolites from fuzzy c‐means clustering of RNA sequencing, proteomics, and metabolomics data.
**Figure S7.** Fuzzy clustering comparison.
**Figure S8.** Postnatal changes in glycolysis and gluconeogenesis in the mouse heart.
**Figure S9.** Postnatal changes in the pyruvate pathway in the mouse heart.
**Figure S10.** Postnatal changes in fatty acid degradation in the mouse heart.

**Appendix S1.** RNA sequencing and differential expression analysis.

**Appendix S2.** Gene set enrichment analysis.

**Appendix S3.** Proteomics.

**Appendix S4.** Metabolomics.

**Appendix S5.** Fuzzy clustering (transcripts, proteins, and metabolites in each cluster) and upstream regulator analysis (Ingenuity pathway analysis; Qiagen).
