# Supplementary material for: Molecular Atlas of Postnatal Mouse Heart Development
Source: J Am Heart Assoc. 2018 Oct 14;7(20):e010378. doi: 10.1161/JAHA.118.010378 (PMC6474944; doi:10.1161/JAHA.118.010378)
Supplement: Supplementary file 1 — Data S1. Supplemental methods. Table S1. Expression of Selected Genes Linked to Cardiac Regeneration and the Postnatal Metabolic Switch Table S2. Proteomics Figure S1. Individual factor maps of the RNA sequencing data principal component (PC) analysis shows perfect separation of sample groups. Figure S2. Top 10 down‐ and upregulated genes in the postnatal mouse heart. Figure S3. Ion channel and control gene expression in the postnatal mouse ventricular tissue. Figure S4. Proteomic changes in neonatal mouse hearts. Figure S5. Individual factor maps of the proteomics data principal component (PC) analysis of sample set 1 (A) and sample set 2 (B). Figure S6. The abundance profiles of all individual transcripts, proteins, and metabolites from fuzzy c‐means clustering of RNA sequencing, proteomics, and metabolomics data. Figure S7. Fuzzy clustering comparison. Figure S8. Postnatal changes in glycolysis and gluconeogenesis in the mouse heart. Figure S9. Postnatal changes in the pyruvate pathway in the mouse heart. Figure S10. Postnatal changes in fatty acid degradation in the mouse heart. [file JAH3-7-e010378-s001.pdf]

SUPPLEMENTAL MATERIAL

Data S1.

Supplemental Methods

Preparation of tissue samples

Mouse pups (strain C57BL/6JOlaHsd, both sexes) were acquired from the experimental animal facility of the University of Helsinki with an internal use licence (KEK14-014) and were used on postnatal days 1, 4, 9 and 23 (P01, P04, P09 and P23, respectively) before weaning. Animals were housed in standard conditions and their handling and all procedures were carried out in accordance with University of Helsinki institutional guidelines, which conform to the National Research Council (US) Guide for the Care and Use of Laboratory Animals.[1] The tissue samples were collected at approximately midday without fasting. All P01-P09 pups had been suckling recently and had milk in their stomachs. Although not weaned, the P23 pups had access to general mouse feed in addition to milk. A total of 92 mice were decapitated, and their ventricles were excised and cut into 3-5 pieces, rinsed in phosphate-buffered saline, snap-frozen in liquid N_2 and stored at -80 °C.

RNA extraction and purification

Ventricular tissue samples from nine mice for each time point from set 2 were randomly pooled together (3 samples / pool) to yield three samples for each time point. The tissue pieces were homogenized in TriZol® (Thermo Fisher Scientific) using Precellys soft tissue beads (Bertin Instruments, Montigny-le-Bretonneux, France) and Precellys24 Homogenizer (Bertin Instruments). Total RNA was extracted using chloroform extraction, precipitated from the aqueous phase with isopropanol, washed using 75% ethanol and dissolved in RNAse free water, whereafter it was purified using RNeasy® Mini Kit (Qiagen) according to manufacturer's instructions. The integrity and quality of the purified RNA was analysed using Agilent 4200 TapeStation (Agilent, Santa Clara, CA, USA).

RNA sequencing

The RNA library for sequencing was prepared with SureSelect Strand-Specific RNA Library Prep Kit (Agilent). Ribosomal RNA was depleted from 1 µg of total RNA using Ribo-Zero Complete Gold rRNA Removal Kit (Illumina, San Diego, CA, USA), whereafter the rRNA-depleted RNA was purified with RNeasy mini Elute columns (Qiagen) and fragmented to generate approximately 300 bp segments. Double-stranded cDNA (ds-cDNA) was then synthesized, 3' ends of the ds-cDNA were adenylated and adaptors ligated and the ds-cDNA was purified with Agencourt AMPure XP beads (Beckman Coulter, Indianapolis, IN, USA). The produced ds-cDNA library was analysed for quantity using Qubit® dsDNA HS kit (Thermo Fisher Scientific) and Qubit® Fluorometer (Thermo Fisher Scientific) and for quality with 2100 Bioanalyzer® (Agilent) using a high-sensitivity chip. Sequencing was then performed as single-end sequencing for read length of 75 bp using Illumina NextSeq Sequencer (Illumina) in one high-output run.

Analysis of RNA sequencing data

Quality of the sequencing data was analysed using FastQC[2] and was considered excellent with average Phred Quality Scores of >30. Quality trimming was applied to the data with the Trimmomatic software.[3] The sample reads were then aligned against the GENCODE M12 (GRCm38.p5) reference genome.[4] STAR-aligner[5] was used in gene-level output mode to produce sorted alignment map files, from which alignment statistics were collected with Qualimap 2.[6] The counts were produced from the STAR output data with the featureCounts software[7] using strand specificity option of 2 due to the reverse-stranded sequencing library. The successfully assigned reads varied from $2x10^6$ to $12x10^6$ reads per sample, which was considered adequate as the duplication level was low.

The quality of the data and differential expression were analysed with DESeq2 software[8] in R[9] environment. The count values were normalized using a geometric mean and sample-wise size factors were estimated to correct for variation in library size. The dispersion of gene-wise values between sample groups was estimated and negative binomial linear model and Wald test were used to produce p values. To optimize p value adjustment, removal of low-expression results and outliers (by Cook's distance) was carried out, whereafter multiple testing adjustment of p values was performed using the Benjamini-Hochberg procedure. Gene ontology (GO)[10,11] enrichment analysis was performed with ranked gene lists using *GO*rilla[12] (available online at http://cbl-gorilla.cs.technion.ac.il).

Tissue homogenization for protein and metabolite extraction

The ventricular tissue samples (Figure 1) were homogenized using a probe sonicator (Rinco Ultrasonics, Arbon, Switzerland) for sample set 1 and FastPrep-24 5G bead homogenizer (MP Biomedicals, Santa Ana, CA, USA) with 1 mm zirconia beads (BioSpec Products, Bartlesville, OK, USA) for sample set 2. The homogenization was done in Milli-Q water and the volume was adjusted according to tissue weight (approximately 1 µL of water per 0.1 mg of tissue, in total 80 - 662 µL). After homogenization, samples were divided into two for metabolomics and proteomics experiments, and stored at -80 °C freezer prior to other pre-treatment steps. Total protein content of the homogenates was measured five times with a Thermo NanoDrop 2000 (Thermo Scientific, Waltham, MA, USA).

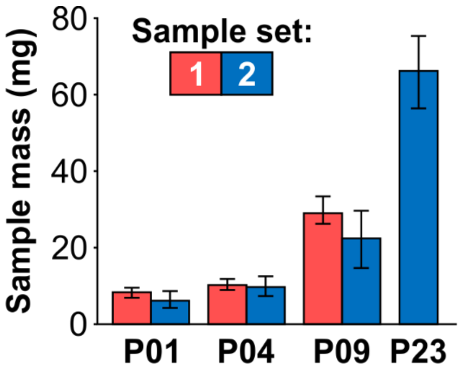

Figure 1. *Weights of the neonatal mouse ventricular samples used for proteomics and metabolomics, shown as mean and range. Samples were from postnatal days 1, 4, 9 and 23 (P01, P04, P09 and P23, respectively).*

Proteomics

The chemicals and solvents were from Sigma-Aldrich (Steinheim, Germany) unless otherwise mentioned. Water was purified with a Milli-Q water purification system (Millipore, Molsheim, France). For liquid chromatography (LC) running buffers, LC-MS grade water was used.

Cell lysis and protein denaturation were done by sonication in 8 mol/L urea (set 1: 200 µL; set 2: 360 µL; Amresco, Solon, OH, USA). Insoluble cell debris was removed by centrifugation at 21000 *g* for 15 min at room temperature, and the samples were diluted to < 1.5 mol/L urea with water (set 1: 900 µL; set 2: 1640 µL). Cysteine residues were reduced with 50 mmol/L dithiothreitol (set 1: 110 µL; set 2: 222 µL; final concentration 5 mmol/L, 20 min incubation in 60 °C) and carbamidomethylated with 150 mmol/L iodoacetamide (set 1: 121 µL; set 2: 246 µL; 20 min incubation in room temperature in dark), after which the pH was adjusted to 7-8 with 200 µL of 1 mol/L ammonium bicarbonate (Fluka Chemie AG, Buchs, Switzerland), and the proteins were digested with 2.5 µg of sequencing grade modified trypsin (Promega, Madison, WI, USA) overnight at 37 °C with shaking. The resulting peptide solution was acidified to pH < 2 with 200 µL of 10 % trifluoroacetic acid and purified using C18 MicroSpin

columns (The Nest Group, Southboro, MA, USA). The columns were conditioned with 3 x 100 µL of acetonitrile, equilibrated with 4 x 100 µL of 1 % acetonitrile and 0.1 % trifluoroacetic acid in water, after which the samples were loaded into the columns in 400 µL aliquots. The washing was done with 4 x 100 µL of 5 % acetonitrile, 0.1 % trifluoroacetic acid in water and the elution with 3 x 100 µL of 50 % acetonitrile and 0.1 % trifluoroacetic acid in water. After the C18 purification, the samples were vacuum centrifuged to dryness and stored in -20 °C.

Before the liquid chromatography-mass spectrometry (LC-MS) analysis, the samples were recovered by sonication in 60 µL of 1 % acetonitrile and 0.1 % trifluoroacetic acid in water. The LC-MS run order was randomized, and pooled quality control samples were used for monitoring system stability throughout the analysis. The injection volume was 4 µL (equivalent to approximately 4 µg of total digested protein, determined based on the total protein content measurement of the homogenate).

All components of the LC-MS setup were from Thermo Scientific (Waltham, MA, USA).The analysis was carried out using an EASY-nLC 1000 coupled to an Orbitrap Elite MS, using Xcalibur version 2.2 SP1 build 48. For the separation, EASY columns were used with the flow rate 300 nL/min at room temperature (pre-column: C18-A1, 100 µm x 2 cm, 5 µm, 120 Å; analytical column: C18-A2, 75 µm x 10 cm, 3 µm, 120 Å). A gradient totaling in 140 min was used: 5 % buffer B (5 min), from 5 to 35 % (120 min), from 35 to 80 % (5 min) , and from 80 to 100 % (1 min) , followed by 9 min column wash with 100 % buffer B (buffer A: 1 % acetonitrile, 0.1 % formic acid, 0.01 % trifluoroacetic acid in water; buffer B: 98 % acetonitrile, 0.1 % formic acid, 0.01 % trifluoroacetic acid in water). Nanospray emitter voltage was set to 3.0 kV (set 1) or 2.8 kV (set 2), based on the current optimized MS tune files. Top20 data-dependent acquisition was applied, in which 20 most intensive precursor ions from a FTMS full scan (*m/z* 300-1700, mass resolution 60000 FWHM) were subjected to CID-MS2 scans (normalized collision energy 35 %) in the ion trap. A 30s dynamic exclusion with a 10 ppm window and a repeat count of 1 was applied.

Protein identification and label-free quantification were done with Andromeda search engine and MaxQuant software, respectively.[13-15] One percent false discovery rate (FDR) filtering was applied on both peptide and protein level. An up-to-date mouse UniProtKB/Swiss-Prot proteome (16,800 proteins restricted to canonical entries, downloaded 2016-08-09) was used as the library,[16] with search mass tolerance 6 ppm in MS1 and 20 ppm in MS2; a maximum of two missed cleavages was allowed. Cysteine carbamidomethylation was set as a static modification, and methionine oxidation as a variable modification. Contaminant and decoy matches were omitted before further data analysis, which was carried out with R[9] using the normalized MS1 intensity (LFQ intensity) values from MaxQuant without further normalization. Protein grouping was applied, and only unique and razor peptides were used for quantification. In addition to base R, the packages ggplot2[17] and FactoMineR[18] were used. The bioinformatic analysis was performed with DAVID 6.8.[19,20]

Metabolomics

Chemicals for two-dimensional gas chromatography-mass spectrometry (GCxGC-MS): Labeled internal standards; dl-valine-d8, succinic acid-d4, glutamic acid-d4 and heptadecanoic acid-d33 as well as retention index standards; undecane, pentadecane, heptadecane, heneicosane, pentacosane and injection standard 4,4,-dibromooctafluorobiphenyl were all from Sigma-Aldrich. Derivatization agent 2% O-methylhydroxylamine hydrochloride in pyridine (MOX reagent) was from Thermo Scientific. N-methyl-N-(trimethylsilyl)trifluoroacetamide (MSTFA) and chlorotrimethylsilane (TMCS) as well as all solvents, n-hexane (Chromasolv® plus for HPLC grade), methanol (LC-MS Ultra CHROMASOLV™) and pyridine (ReagentPlus® grade), were from Sigma-Aldrich.

Chemicals for LC-MS: All aqueous solutions were prepared utilizing ultrapure water purified with Milli-Q system (Millipore, Molsheim, France). LC-MS Chromasolv™ grade methanol and isopropanol were from Sigma-Aldrich (Steinheim, Germany), and formic acid from Merck

(Darmstadt, Germany). The standard mixture contained L-lysine-d4, palmitidic acid-d31, verapamil, propranolol and ibuprofen (from Sigma-Aldrich), 1-hexadecanoyl(d31)-2-(9Z-octadecenoyl)-sn-glycero-3-phosphocholine (PC(16:0 d31/18:1)), 1-hexadecanoyl(d31)-2-(9Z-octadecenoyl)-sn-glycero-3-[phospho-rac-(1-glycerol)] (PG(16:0 d31/18:1)), 1-heptadecanoyl-2-hydroxy-sn-glycero-3-phosphocholine (LysoPC(17:0)) and 27-hydroxycholesterol-d6 (from Avanti Polar Lipids; Alabaster, AL, USA) and pregnenolone-d4 (from Steraloids; Newport, RI, USA).

Sample preparation for GCxGC-MS analysis: Tissue homogenates were thawed on ice and 25 µL of homogenate was transferred into a new Eppendorf-tube. Methanol (410 µL) containing labeled internal standards (0.9-4 µg/mL) was added to the samples to precipitate the proteins. The samples were vortexed, sonicated for 5 min, and incubated on ice for 30 min, whereafter the extracts were centrifuged for 5 min (14 338 *g*, 4°C) and 180 µl aliquots of supernatants were transferred into vials and evaporated to dryness under nitrogen flow. The metabolites were then converted into their methoxime and trimethylsilyl (TMS) derivatives by automated two-step derivatization. First, 25 µL of MOX was added to the residue, and the mixture was incubated for 1 h at 45 °C. Next, 25 µL of MSTFA including the retention index standards was added, and the mixture was incubated for 1 h at 45 °C. Finally, hexane including injection standard (50 µL) was added to the mixture immediately prior to injection. Quality control (QC) samples were prepared by pooling P1, P4, and P10 tissue samples, which were prepared similarly as described above. All metabolomics samples and pooled QC-samples were randomized before the sample pretreatment and the analysis.

Sample preparation for LC-MS analysis: To 25 µL aliquots of sample homogenates, 5 µL of internal standard mixture (containing 4-20 µg/mL of PC(16:0 d31/18:1), PG(16:0 d31/18:1), LysoPC(17:0), 27-hydroxycholesterol-d6, verapamil and propranolol, L-Lysine-d4, palmitic acid-d31, ibuprofen and pregnenolone-d4) and 100 µL of methanol were added, whereafter the samples were vortexed and incubated on ice for 30 min to precipitate the proteins. The samples were then centrifuged for 10 min at 7 378 *g* at room temperature, and supernatants were transferred to vials and analyzed in random order.

GCxGC-MS analysis of polar metabolites: Polar metabolites were analyzed by GC×GC–MS at Steno Diabetes Center (Gentofte, Denmark) as described earlier.[21] The analyses were performed with a LECO Pegasus 4D instrument consisting of an Agilent 7890 gas chromatograph equipped with a split/splitless injector (Agilent Technologies, Santa Clara, CA), a cryogenic dual-stage modulator and a time-of-flight mass spectrometer (Leco Corp., St. Joseph, MI, USA). Instrumentation included a multipurpose sampler from Maestro software (Gerstel, Mülheim an der Ruhr, Germany), which was used for derivatization and sample introduction. A 10 m × 0.18 mm I.D. RTXi-5MS column with film thickness of 0.2 µm (Restek Corp., Bellefonte, PA, USA) was used as the first column and a 1.5 m × 0.1 mm I.D. BPX-50 column with a film thickness of 0.1 µm (SGE Analytical Science, Austin, TX, USA) as the second column. A deactivated fused silica retention gap column (1.7 m × 0.53 mm I.D., Agilent technologies, Santa Clara, CA) was connected in front of the first column. The injector temperature was 240 °C and the samples (1 µL) were injected using pulsed splitless mode with a splitless period of 90 s. High-purity helium (99.9999%, Yara Praxair) was used as the carrier gas in a constant-pressure mode with initial pressure of 40 psig. The first column oven temperature program was as follows: 50 °C in isothermal mode for 2 min, from 50 °C to 240 °C at 7 °C/min, from 240 °C to 300°C at 25 °C/min, and kept at 300 ° for 3 min. The second dimension column oven temperature was maintained constantly 20 °C higher than the first column oven and the modulation time was 4 s. The temperature of the transfer line was 260 °C. The temperature of the electron impact ion source was 200 °C and the electron energy 70 eV. The mass range was from 45 to 700 m/z and 100 spectra/s were measured.

LC-MS Analysis: All experiments were performed with a Thermo Orbitrap Fusion high resolution mass spectrometer equipped with a Thermo Dionex Ultimate 3000 UHPLC (both from Thermo Fisher Scientific). The column was Waters Acquity BEH C18 (2.1 x 100 mm, 1.7 µm). The separation was performed using a 15-min linear gradient from 95% A (0.1% formic

acid in Milli-Q water) to 100% B (0.1% formic acid in methanol) followed by a 10-min isocratic period of 100% B and a 5-min equilibration to initial conditions. For the sample set 2, a minor modification was done for the LC method so that the starting eluent was 100% A (0.1% formic acid and 5% methanol in MQ water). The flow rate was 0.3 mL/min, total run time 30 min, the column temperature 25 °C, and the sample tray was cooled to 10 °C. The injection volume was 2 µL for the first sample set and 3 µL for the second sample set. In the FTMS full scan the mass resolution was 120 000 FWHM and the mass range from m/z 100 to m/z 1000. The spectra were collected in centroid mode with a duty cycle time of 0.6 s, applying internal calibration (Easy-IC with fluorantine). Heated electrospray, with a spray voltage of 3.0 kV in the positive ion mode was applied for ionization. The other source parameters were: sheath gas 30 arb, aux gas 10 arb, ion transfer tube temperature 333 °C and vaporizer temperature 317 °C. For identification purposes MS/MS spectra were collected applying quadrupole isolation window of 1, HCD fragmentation energies of 25% and 40% and mass resolution of 30 000 FWHM.

GCxGC-MS data processing and analysis: ChromaTOF version 4.71 (LECO Corporation, St. Joseph, MI) was used for the raw data processing. Automatic peak detection and mass spectrum deconvolution were performed using a peak width of 0.2 s and a peak signal-to-noise (S/N) value threshold of 100. The data files obtained by the ChromaTOF software were exported to text files and *Guineu* software[22] was used for the compound alignment. The column bleed was removed and only the TMS-derivatized peaks (73 m/z ion in the spectra) were selected for further data processing. The peak areas from total ion chromatograms (TIC) were used for most of the compounds. For a pre-selected set of compounds (see Supplemental results, Table 2), the peak areas of the selected ions were monitored and quantified based on external calibration curves. The linear retention indices (RIs) were calculated based on the retention times of the retention index standards (*n*-alkanes). The alignment of the data was performed based on RIs, first and second dimension retention times as well as electron ionization (EI) spectral matches. Only the metabolites detected in more than 10% of the analyzed samples were included in the dataset.

The metabolites were identified tentatively by comparing the measured mass spectra and the RIs to those in NIST 2014 or in-house libraries. In addition, Golm metabolome database[23] and Fiehn library[24] was used for the further identification of interesting features. Identification annotations are based on the recommended identification criteria by metabolite standard initiative.[25] Identification level 1 is annotated to compounds identified against standard compounds. Level 2 identifications are annotated to compounds, whose spectral match is > 850 and RI difference < 30 compared to respective values in NIST 2014 or in-house library. Level 3 is compound class specific annotation for the findings, whose spectral match is >800 against NIST 2014 library and are additionally searched against other library (Golm metabolome database or Fiehn library). Level 4 spectral match is below these criteria and has been annotated as unknown.

Data processing was performed with R 3.1.2.[9] The data were filtered further with the criteria that the peak should be found in 75% of the samples in at least one of the sample groups (P01, P04, P09, P23). Principal component analysis (PCA) as well as t-tests with FDR-correction (Benjamini and Hochberg) were performed to both sample sets separately. The features in the two separate sample sets were aligned and combined according to maximum dot product of spectra, retention index difference ≤ 10 and second retention time ≤ 0.25 s, respectively. Combined data was explored with linear mixed effect model (LME),[26] where the first variable was the postnatal age and the second was the dataset. P01 estimate was set to 0 with variance one and other sample groups were compared to the P01.

LC-MS data processing and analysis: MzMine2[27] was used for the data processing, and the following functions were applied: mass detection (centroid algorithm), chromatogram builder, chromatogram deconvolution (local minimum search algorithm), deisotoping, alignment (joint aligner), filtering (number of found), and cap filling. Peak intensity was normalized to the intensity of internal standard LysoPC(17:0) and to the mass of the tissue sample. Differences

between the sample groups of the individual data sets were searched with one-way ANOVA followed by Tukey`s HSD *post-hoc* test using R and metadar package. Fold change values were calculated and p-values adjusted with the FDR correction (Benjamini and Hochberg). Features were also combined with LME model, with the following criteria: a maximum mass difference of 2 ppm and a maximum retention time difference of 0.35 min. If multiple matching features were found with several retention times, correct alignments were confirmed manually. MS/MS spectra were collected for all the features from the sample set 1 showing statistical difference ($q < 0.01$) in ANOVA. The features showing significant differences also with LME model were identified by searching the measured spectra against existing spectral databases or repositories; mzCloud (https://www.mzcloud.org/), NIST 2014 MS/MS (http://chemdata.nist.gov/), HMDB[28], MoNA (http://mona.fiehnlab.ucdavis.edu). In addition, *in silico* database LipidBlast[29] and molecular structure database search tool CSI:Finger ID[30] or known group-specific fragmentation of lipid species was used in the identification. Phospholipids were identified and annotated based on the exact mass information and known polar head group fragments of phospholipids. These are annotated with phospholipid class, the number of carbons and double bonds in the fatty acid chains (e.g. PC(32:0)).

Metabolite set enrichment analysis (MSEA)[31] was performed to all significantly changed ($q<0.01$) identified metabolites in LME model. Pathway-associated metabolite set library was applied and all compounds from the reference metabolite library were selected for the enrichment analysis.

Fuzzy clustering

To compare and integrate the results of the three different omics analyses, unsupervised pattern classification was carried out using fuzzy c-means clustering,[32-34] in which the transcripts, proteins, and metabolites were assigned into one or more clusters based on their abundance pattern. The analysis was carried out using the C-means clustering package in R[9] with fixed numbers of clusters (7 for transcriptomics and proteomics, 5 for metabolomics) and the fuzziness value was determined according to the method described in [34]. The input data for fuzzy c-means clustering was limited to transcripts, proteins, and metabolites that were significantly different in abundance in one or more of the comparisons in sample set 2.

Fuzzy clusters were analyzed with DAVID 6.8[19,20] for GO term[10,11] and KEGG[35] pathway enrichment using the recommended settings. Upstream regulator analysis was performed with IPA (QIAGEN Inc., https://www.qiagenbioinformatics.com/products/ingenuity-pathway-analysis) with the standard settings. Upstream regulators of the classes “transcription regulator”, “microRNA”, and “chemical - endogenous mammalian” with $p < 0.01$ were included in the analysis.

Cardiomyocyte proliferation and viability

Primary cultures of rat neonatal ventricular cardiomyocytes and fibroblasts were prepared from 1–3 day old Wistar rats as described earlier.[36] Briefly, animals were decapitated and ventricles excised and enzymatically digested with 2 mg/mL collagenase type 2 and 2 mg/mL pancreatin (in a buffer containing 100 mmol/L NaCl, 10 mmol/L KCl, 1.2 mmol/L KH_2PO_4, 4.0 mmol/L $MgSO_4$, 50 mmol/L taurine, 20 mmol/L glucose, 10 mmol/L 4-(2-hydroxyethyl)-1-piperazineethanesulfonic acid (HEPES), 100 U/mL of penicillin, and 100 µg/mL of streptomycin) for 1–2 h at 37 °C with gentle shaking. The cells were collected by centrifugation and the majority of non-cardiomyocytes removed with a 1-h pre-plating in DMEM/F12 supplemented with 10% fetal bovine serum (FBS; from Gibco), 100 U/mL of penicillin, and 100 µg/mL of streptomycin (Gibco) in cell culture incubator (37 °C, 5% CO_2, humidified atmosphere), whereafter the unattached cells representing enriched cardiomyocytes were plated at 40 000 cells/well on 96-well plates (Corning® Costar®). On the following day, the medium was changed to complete serum-free medium (CSFM; DMEM/F-12 supplemented with 2.5 mg/ml bovine serum albumin, 5 µg/ml insulin, 5 µg/mL transferrin, 5 ng/mL selenium,

2.8 mM sodium pyruvate, 0.1 nM triiodo-L-thyronine (T3), 100 U/mL of penicillin, and 100 µg/mL of streptomycin), and the cells were let to adapt for 24 h prior to drug treatments.

In order to analyze cell viability, the cardiomyocyte cultures were treated with hymeglusin or simvastatin (from Santa Cruz and Sigma-Aldrich) at concentrations of 1-100 µmol/L (µM) for 24 h in CSFM and cell viability was analyzed using the 3-(4,5-dimethylthiazol-2-yl)-2,5-diphenyltetrazolium bromide (MTT) assay as described previously.[37] Briefly, 0.5 mg/mL MTT was added to the medium for 2 hours (incubation at 37 °C, 5% CO_2, humidified atmosphere), whereafter the medium was removed and formazan crystals dissolved in DMSO (200 µL/well). The absorbance was then measured at 550 nm with absorbance at 650 nm subtracted as background. Data are presented as percentage of control, mean+SEM from three independent experiments carried out in triplicate.

To quantify cell proliferation, the cardiomyocyte cultures were exposed to hymeglusin (10 µM or 100 µM) or simvastatin (1 µM or 10 µM) in CSFM (±5% FBS) containing 10 µM bromodeoxyuridine (BrdU; from Sigma-Aldrich) for 24 h. Cells were then fixed with 4% paraformaldehyde and stained for cardiac α-actinin (A7811, Sigma-Aldrich; 1:600) and BrdU (ab6326, Abcam; 1:250). The secondary antibodies used were AlexaFluor® 488 conjugated anti-mouse (A-11029, Thermo Scientific; 1:200) and AlexaFluor® 647 conjugated anti-rat (A-21247, Thermo Scientific; 1:200), and 4',6-diamidino-2-phenylindole (DAPI, 5 µg/mL) was used to stain DNA. The cells were imaged and analyzed using a CellInsight CX5 High Content Screening Platform (Thermo Scientific) equipped with an Olympus UPlanFL N 10x/0.3 objective as described previously.[38] Non-cardiomyocytes were excluded based on absence of α-actinin staining, and the percentage of $BrdU^+$ cardiomyocyte nuclei was quantified.

Statistical analysis of cell viability and proliferation data was carried out with IBM SPSS Statistics version 24 using Welch ANOVA followed by the Games-Howell post hoc test. The level of statistical significance was set at $p < 0.05$.

Supplemental Metabolomics Results

GCxGC-MS analysis:

The repeatability of the analytical method was evaluated by determining the peak areas of the internal and injection standards (Table 1) added to all heart samples (n=44 in sample set 1 and n=39 in sample set 2) and the peak areas of the quantified compounds (Table 1, Figure 2) in pooled heart samples (n=10 in sample set 1 and 2) used as quality control (QC) samples. The samples were pretreated and analyzed with GCxGC-MS in random order and quantified using external calibration. The relative standard deviation (RSD%) values for the internal and injection standards were between 8 and 21% indicating good repeatability of the GCxGC-MS method. The repeatability of the quantified compounds was typically below 30% (RSD%), which is considered acceptable in metabolomics analysis.

***Table 1**. Relative standard deviations (RSD %) of the internal (int. st.) (succinic acid-d4 and heptadecanoic acid-d33) and injection standards (inj. st.) (4,4'-dibromooctafluorobiphenyl) intensities in heart sample sets 1 (n=44) and 2 (n=39), and quantified compounds in pooled QC heart samples sets 1 (n=10) and 2 (n=10) measured with GCxGC-MS. (TMS = Trimethylsilyl).*

**Compounds**	**RSD%**	
**Internal and injection standards:**	**Sample set 1**	**Sample set 2**
succinic acid-d4 (int, st.)	11	21
heptadecanoic acid-d33 (int. st.)	9	8
4,4′-dibromooctafluorobiphenyl (inj. st.)	14	14
**Quantified compounds**		
3-Hydroxybutyric acid, 2TMS	16	14
Alanine, 2TMS	12	18
Arachidonic acid, TMS	50	28
Aspartic acid, 3TMS	62	78
Cholesterol, TMS	12	17
Citric acid, 4TMS	11	26
Fumaric acid, 2TMS	5	21
Glutamic acid, 3TMS	4	5
Glycine, 3TMS	19	37
Isoleucine, 2TMS	23	24
Leucine, 2TMS	31	20
Linoleic acid, TMS	20	13
Malic acid, 3TMS	7	7
Oleic acid, TMS	17	9
Palmitic acid, TMS	19	14
Proline, 2TMS	45	23
Serine, 3TMS	35	30
Stearic acid, TMS	26	19
Succinic acid, 2TMS	14	16
Threonine, 3TMS	24	22
Valine, 2TMS	3	7

The metabolites in the heart tissue samples were analyzed as their TMS derivatives and the peak areas were used in determining the relative abundances of the detected features. After the first data preprocessing steps, the GCxGC-MS datasets of the sample set 1 and 2 contained 760 and 771 metabolic features, respectively. After filtering, the total numbers of features were 347 and 443. These features were further analyzed with t-test applying FDR correction (Supplemental Dataset S4). The data from the two separate sample sets were combined based on spectral alignment, retention index and second retention time difference. The combined dataset resulted in 328 features, which were analyzed with LME model resulting in total 162 metabolic features with statistical significance (q <0.01) in at least one of the group comparisons (P01 vs. P04, P01 vs. P09, P01 vs. P23; Supplemental Dataset S4).

Principal component analysis of all samples and pooled quality control samples from the sample set 2 shows some grouping of the samples based on postnatal age (Figure 2). The P01, P04, and P09 sample groups cannot be totally distinguished from each other with the two principal components. However, P23 is totally separated from the other sample groups and the QC samples clustered among all sample groups in the individual factor maps plot (Figure 2). Even though the QC samples show some variation in the direction of principal component 1, the variation in the direction of principal component 2 is explained more with the postnatal age.

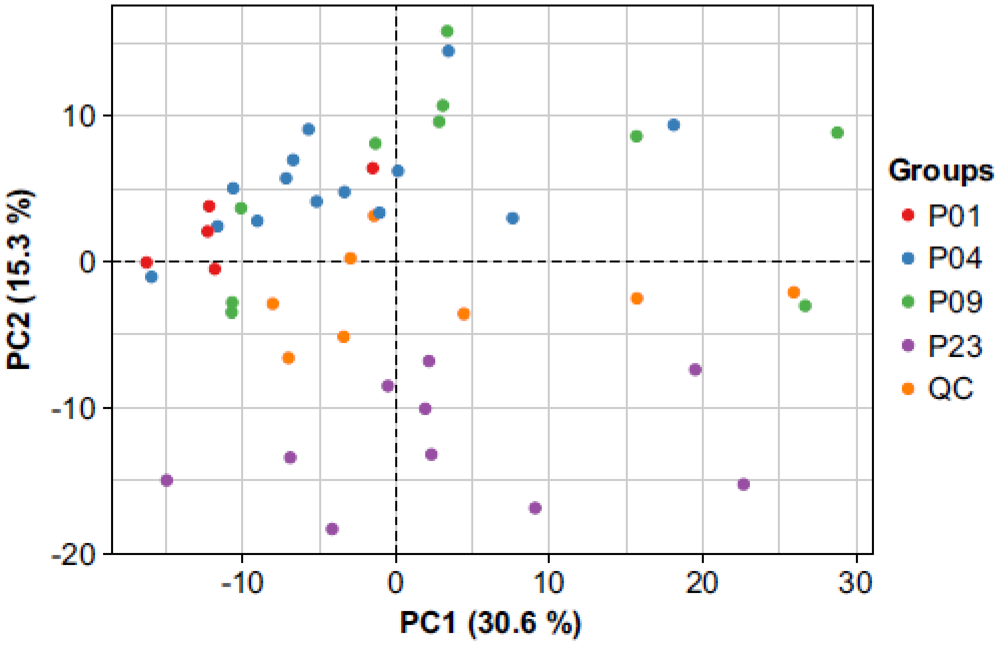

Figure 2. *Individuals factor map of the GCxGC-MS data principal component (PC) analysis of sample set 2. QC, quality control.*

LC-MS analysis

The analytical performance of the LC-MS method using the orbitrap mass spectrometer was evaluated in terms of repeatability of peak intensities (peak area), retention times and mass accuracies of the internal standards added to the heart tissue samples (n=44 in sample set 1 and n=39 in sample set 2). The samples were pretreated and analyzed in random order. The RSD% for the peak intensities without normalization and after normalization against LysoPC(17:0) were below 25%, which is considered acceptable in metabolomics. Furthermore, no systematic trend in signal intensities were observed. The mass errors between exact mass (calculated) and accurate masses (mean, measured) were below 1 ppm indicating excellent mass accuracies. The differences of mass accuracies between the sample sets 1 and 2 were below 0.1 ppm and those of retention times below 0.25 min. Based on these

values the maximum mass difference of 2 ppm and maximum retention time difference 0.35 min were used as limiting values for combining the heart tissue sample sets 1 and 2 with LME model.

Table 2. *Exact masses, accurate masses, retention times (RT), mass errors, and relative standard deviations (RSD%) of the internal standards in heart sample sets 1 (n=44) and 2 (n=39) and in pooled quality control (QC) samples in sample sets 1 (n=10) and 2 (n=10) measured with LC-MS using orbitrap mass spectrometer.*

Compound	Exact mass	Accurate mass	RT (min)	Mass error (ppm)	RSD% intensity	RSD% normalized intensity
**Sample set 1**						
LysoPC(17:0)	510.35542	510.35524	15.84	0.35	24.8	0.0
Propranolol	260.16451	260.16434	9.48	0.67	25.0	17.7
L-Lysine-d4	151.13790	151.13794	0.78	-0.25	23.4	21.2
Verapamil	455.29043	455.29018	10.15	0.56	16.3	15.4
**Sample set 2**						
LysoPC(17:0)	510.35542	510.35522	16.01	0.38	11.7	0.0
Propranolol	260.16451	260.16431	9.24	0.78	12.9	14.5
L-Lysine-d4	151.13790	151.13793	0.78	-0.23	22.8	24.2
Verapamil	455.29043	455.29019	9.90	0.52	12.9	14.5

After preprocessing, the LC-MS analysis of the sample sets 1 and 2 comprised 5591 and 9043 metabolic features, respectively. The detected features from the sample sets were analyzed separately with ANOVA. The q values and fold changes are presented in Dataset S4. The features from individual datasets were further combined based on retention time and mass-to-charge ratio difference observed with the internal standards in the sample sets 1 and 2 (Table 2) and the combined dataset containing 3398 features was analyzed with the LME model. In total 805 metabolic features displayed statistical significance ($q < 0.01$) in at least one of the group comparisons (P01 vs. P04, P01 vs. P09, P01 vs. P23; Dataset S4).

Figure 3 shows the individuals factor map of principal component analysis the heart samples and pooled QC samples from the sample set 2 analyzed by LC-MS. The QC samples clustered closely together between the samples, indicating that the variation observed within the sample groups reflects the characteristics of the samples and is not due to the analysis.

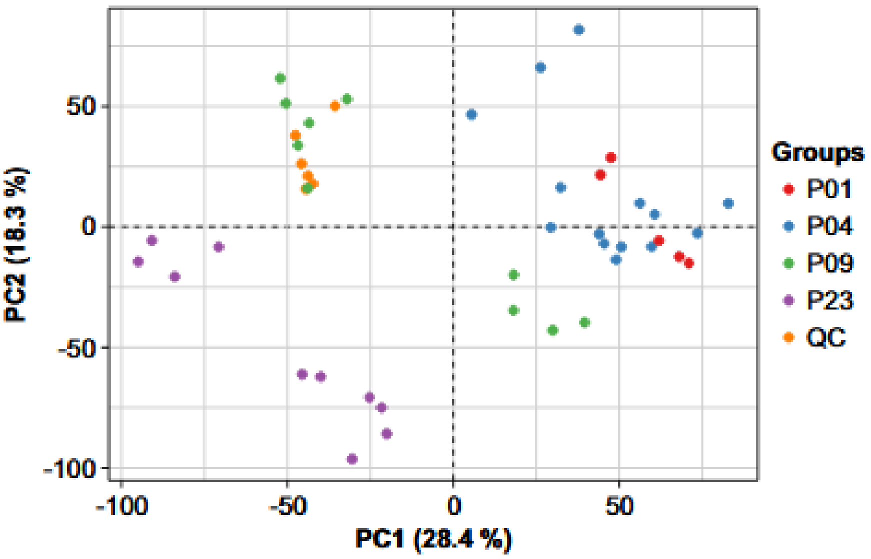

Figure 3. *Individuals factor map of the LC-MS data principal component (PC) analysis of sample set 2. QC, quality control.*

Dataset Legends (see Excel files):

Dataset S1. RNA sequencing and differential expression analysis.

Dataset S2. Gene set enrichment analysis (GSEA).

Dataset S3. Proteomics.

Dataset S4. Metabolomics.

Dataset S5. Fuzzy clustering (transcripts, proteins and metabolites in each cluster) and upstream regulator analysis (IPA).

Supplemental References:

1. National Research Council (US) Committee for the Update of the Guide for the Care and Use of Laboratory Animals. 8th edition, 2011. Available online at https://www.nap.edu/catalog/12910/guide-for-the-care-and-use-of-laboratory-animals-eighth. Accessed April 18, 2018.

2. Andrews S. FastQC - A quality control tool for high throughput sequence data. Available online at: www.bioinformatics.babraham.ac.uk/projects/fastqc/. Accessed April 18, 2018.

3. Bolger AM, Lohse M, Usadel B. Trimmomatic: A flexible trimmer for illumina sequence data. *Bioinformatics*. 2014;30:2114-2120.

4. Mudge JM, Harrow J. Creating reference gene annotation for the mouse C57BL6/J genome assembly. *Mamm Genome*. 2015;26:366-378.

5. Dobin A, Davis CA, Schlesinger F, Drenkow J, Zaleski C, Jha S, Batut P, Chaisson M, Gingeras TR. STAR: Ultrafast universal RNA-seq aligner. *Bioinformatics*. 2013;29:15-21.

6. Okonechnikov K, Conesa A, Garcia-Alcalde F. Qualimap 2: Advanced multi-sample quality control for high-throughput sequencing data. *Bioinformatics*. 2016;32:292-294.

7. Liao Y, Smyth GK, Shi W. featureCounts: An efficient general purpose program for assigning sequence reads to genomic features. *Bioinformatics*. 2014;30:923-930.

8. Love MI, Huber W, Anders S. Moderated estimation of fold change and dispersion for RNA-seq data with DESeq2. *Genome Biol*. 2014;15:550.

9. R Core Team. R: A language and environment for statistical computing. 2017;3.4.3. Available from: https://www.R-project.org. Accessed April 18, 2018.

10. The Gene Ontology Consortium. Expansion of the gene ontology knowledgebase and resources. *Nucleic Acids Res.* 2017;45:D331-D338.

11. Ashburner M, Ball CA, Blake JA, et al. Gene ontology: Tool for the unification of biology. the gene ontology consortium. *Nat Genet.* 2000;25:25-29.

12. Eden E, Navon R, Steinfeld I, Lipson D, Yakhini Z. GOrilla: A tool for discovery and visualization of enriched GO terms in ranked gene lists. *BMC Bioinformatics*. 2009;10:48.

13. Cox J, Neuhauser N, Michalski A, Scheltema RA, Olsen JV, Mann M. Andromeda: a peptide search engine integrated into the MaxQuant environment. *J.Proteome Res.* 2011;10:1794-1805.

14. Cox J, Mann M. MaxQuant enables high peptide identification rates, individualized p.p.b.-range mass accuracies and proteome-wide protein quantification. *Nat Biotechnol.* 2008;26: 1367-1372.

15. Cox J, Hein MY, Luber CA, Paron I, Nagaraj N, Mann M. Accurate proteome-wide label-free quantification by delayed normalization and maximal peptide ratio extraction, termed MaxLFQ. *Mol Cell Proteomics*. 2014;13:2513-2526.

16. The UniProt Consortium. UniProt: the universal protein knowledgebase. *Nucleic Acids Res.* 2017;45:D158-D169.

17. Wickham H. ggplot2: Elegant Graphics for Data Analysis. 2009; Springer, New York.

18. Lê S, Josse J, Husson F. FactoMineR: An R package for multivariate analysis. *Journal of Statistical Software.* 2008;25:1-18.

19. Huang da W, Sherman BT, Lempicki RA. Bioinformatics enrichment tools: Paths toward the comprehensive functional analysis of large gene lists. Nucleic Acids Res. 2009;37:1-13.

20. Huang da W, Sherman BT, Lempicki RA. Systematic and integrative analysis of large gene lists using DAVID bioinformatics resources. *Nat Protoc.* 2009;4:44-57.

21. Hartonen M, Mattila I, Ruskeepää A, Oresic M, Hyötyläinen T. Characterization of cerebrospinal fluid by comprehensive two-dimensional gas chromatography coupled to time-of-flight mass spectrometry. *Journal of Chromatography A*. 2013;1293:142-149.

22. Castillo S, Mattila I, Miettinen J, Oresic M, Hyötyläinen T. Data analysis tool for comprehensive two-dimensional gas chromatography/time-of-flight mass spectrometry. *Anal Chem*. 2011;83:3058-3067.

23. Kopka J, Schauer N, Krueger S, Birkemeyer C, Usadel B, Bergmüller E, Dörmann P, Weckwerth W, Gibon Y, Stitt M, Willmitzer L, Fernie AR, Steinhauser D. GMD@CSB.DB: The golm metabolome database. *Bioinformatics*. 2005;21:1635-1638.

24. Kind T, Wohlgemuth G, Lee DY, Lu Y, Palazoglu M, Shahbaz S, Fiehn O. FiehnLib: Mass spectral and retention index libraries for metabolomics based on quadrupole and time-of-flight gas chromatography/mass spectrometry. *Anal Chem*. 2009;81:10038-10048.

25. Sumner LW, Amberg A, Barrett D, et al. Proposed minimum reporting standards for chemical analysis: Chemical analysis working group (CAWG) metabolomics standards initiative (MSI). *Metabolomics*. 2007;3:211-221.

26. Bates D, Mächler M, Bolker BM, Walker SC. Fitting linear mixed-effects models using lme4. *J Stat Software*. 2015;67:1-48.

27. Pluskal T, Castillo S, Villar-Briones A, Oresic M. MZmine 2: Modular framework for processing, visualizing, and analyzing mass spectrometry-based molecular profile data. *BMC Bioinformatics*. 2010;11:395.

28. Wishart DS, Tzur D, Knox C, et al. HMDB: The human metabolome database. *Nucleic Acids Res*. 2007;35:D526.

29. Kind T, Liu K-, Lee DY, Defelice B, Meissen JK, Fiehn O. LipidBlast in silico tandem mass spectrometry database for lipid identification. *Nature Methods*. 2013;10:755-758.

30. Dührkop K, Shen H, Meusel M, Rousu J, Böcker S. Searching molecular structure databases with tandem mass spectra using CSI:FingerID. *Proc Natl Acad Sci U S A*. 2015;112:12580-12585.

31. Xia J, Wishart DS. MSEA: A web-based tool to identify biologically meaningful patterns in quantitative metabolomic data. Nucleic Acids Res. 2010;38:W71-W77.

32. Bezdek JC. Pattern Recognition with Fuzzy Objective Function Algorithms. Springer US; 2013.

33. Dembele D, Kastner P. Fuzzy C-means method for clustering microarray data. *Bioinformatics*. 2003;19:973-980.

34. Schwammle V, Jensen ON. A simple and fast method to determine the parameters for fuzzy c-means cluster analysis. *Bioinformatics*. 2010;26:2841-2848.

35. Kanehisa M, Furumichi M, Tanabe M, Sato Y, Morishima K. KEGG: New perspectives on genomes, pathways, diseases and drugs. *Nucleic Acids Res.* 2017;45:D353-D361.

36. Välimäki MJ, Tölli MA, Kinnunen SM, Aro J, Serpi R, Pohjolainen L, Talman V, Poso A, Ruskoaho HJ. Discovery of small molecules targeting the synergy of cardiac transcription factors GATA4 and NKX2-5. *J Med Chem*. 2017;60:7781-7798.

37. Talman V, Tuominen RK, Boije af Gennäs G, Yli-Kauhaluoma J, Ekokoski E. C1 domain-targeted isophthalate derivatives induce cell elongation and cell cycle arrest in HeLa cells. *PLoS One*. 2011;6:e20053.

38. Karhu ST, Välimäki MJ, Jumppanen M, Kinnunen SM, Pohjolainen L, Leigh RS, Auno S, Földes G, Boije Af Gennäs G, Yli-Kauhaluoma J, Ruskoaho H, Talman V. Stem cells are the most sensitive screening tool to identify toxicity of GATA4-targeted novel small-molecule compounds. *Arch Toxicol.* 2018, Jul 9. doi: 10.1007/s00204-018-2257-1 [Epub ahead of print].

Table S1. Expression of selected genes linked to cardiac regeneration and the postnatal metabolic switch. The RNAseq data have been normalized to P01 and are presented as mean ± SEM. Bold values indicate statistically significant ($q < 0.01$) change compared to at least one time point. The gene names and detailed statistics are available in Dataset 1.

	**P01**	**P04**	**P09**	**P23**
Metabolic switch				
Hif1a	**1.00 ± 0.043**	**1.01 ± 0.033**	**0.95 ± 0.006**	**0.69 ± 0.031**
Hand1	1.00 ± 0.179	0.24 ± 0.120	0.00 ± 0.000	0.00 ± 0.000
Ppara	**1.00 ± 0.028**	**1.14 ± 0.043**	**1.10 ± 0.021**	**2.04 ± 0.061**
PPard	**1.00 ± 0.061**	**0.94 ± 0.092**	1.64 ± 0.047	**2.33 ± 0.346**
Pparg	1.00 ± 0.069	**0.90 ± 0.050**	1.34 ± 0.136	**1.63 ± 0.138**
Mitochondrial turnover				
Park2	1.00 ± 0.068	**0.64 ± 0.067**	0.90 ± 0.020	**1.10 ± 0.101**
Pink1	**1.00 ± 0.040**	**0.59 ± 0.014**	**1.12 ± 0.020**	**2.34 ± 0.037**
Cardiomyocyte proliferation				
Nrg1	1.00 ± 0.395	1.01 ± 0.021	0.71 ± 0.050	0.79 ± 0.093
Erbb2	**1.00 ± 0.012**	**0.66 ± 0.026**	**0.47 ± 0.020**	**0.25 ± 0.022**
Gata4	**1.00 ± 0.014**	**1.08 ± 0.040**	**0.86 ± 0.034**	**0.71 ± 0.030**

Table S2. Proteomics. Log2 fold change and q values for proteins mentioned in the text.

Statistically significant changes (q < 0.01) are indicated in **bold**	Upregulated	Downregulated	q-value < 0.01	q-value < 0.05	Not significant

Accession	Gene name	Protein name	P01 → P04				P01 → P09				P04 → P09				P01 → P23		P04 → P23		P09 → P23	
			Set 1		Set 2		Set 1		Set 2		Set 1		Set 2		Set 2		Set 2		Set 2	
			log2 fold	q-value	log2 fold	q-value	log2 fold	q-value	log2 fold	q-value	log2 fold	q-value	log2 fold	q-value	log2 fold	q-value	log2 fold	q-value	log2 fold	q-value
P17182	Eno1	Alpha-enolase	-0.21	9.76E-02	**-0.71**	**8.84E-10**	**-0.43**	**4.52E-05**	**-1.46**	**4.68E-11**	-0.22	1.67E-01	**-0.75**	**3.81E-05**	**-1.84**	**7.60E-12**	**-1.13**	**9.90E-09**	-0.38	4.63E-01
P12382	Pfkl	ATP-dependent 6-phosphofructokinase, liver type	**-1.25**	**8.89E-09**	-0.39	1.69E-02	**-2.43**	**3.12E-11**	**-0.65**	**5.98E-05**	-1.17	1.50E-02	-0.26	6.68E-01	**-1.10**	**5.77E-09**	**-0.71**	**6.43E-04**	-0.45	1.89E-01
P09411	Pgk1	Phosphoglycerate kinase 1	-0.31	4.09E-02	**-0.49**	**1.65E-05**	**-0.78**	**1.83E-07**	**-0.64**	**2.39E-07**	**-0.47**	**8.75E-03**	-0.15	9.87E-01	**-0.53**	**2.05E-06**	-0.04	1.00E+00	0.11	9.98E-01
Q8CAQ8	Immt	MICOS complex subunit Mic60	**0.26**	**6.40E-03**	0.19	6.83E-02	**0.39**	**1.18E-06**	**0.45**	**3.76E-08**	0.14	1.67E-01	**0.26**	**1.10E-03**	**0.98**	**7.60E-12**	**0.78**	**1.10E-11**	**0.52**	**3.11E-11**
P67778	Phb	Prohibitin	**0.43**	**6.00E-04**	0.21	2.09E-01	**0.59**	**2.14E-07**	**0.37**	**4.80E-04**	0.16	2.74E-01	0.16	4.10E-01	**0.54**	**5.45E-08**	**0.33**	**2.33E-04**	0.17	1.96E-01
Q99M87	Dnaja3	DnaJ homolog subfamily A member 3, mitochondrial	0.55	5.09E-01	0.33	1.64E-01	**1.09**	**5.00E-04**	**0.78**	**1.23E-07**	0.54	1.22E-01	**0.45**	**1.21E-03**	**1.23**	**7.60E-12**	**0.90**	**1.10E-11**	**0.44**	**1.22E-05**
P09671	Sod2	Superoxide dismutase [Mn], mitochondrial	0.88	8.29E-01	0.39	2.05E-01	**1.85**	**5.84E-04**	**0.77**	**2.33E-05**	0.97	5.78E-02	0.38	6.57E-02	**1.95**	**7.60E-12**	**1.56**	**1.10E-11**	**1.18**	**3.11E-11**
P20108	Prdx3	Thioredoxin-dependent peroxide reductase, mitochondrial	0.72	4.91E-01	-0.07	1.00E+00	**1.40**	**2.52E-04**	0.35	9.51E-02	0.67	8.27E-02	0.42	5.73E-02	**1.26**	**7.60E-12**	**1.33**	**1.10E-11**	**0.90**	**3.91E-11**
P99029	Prdx5	Peroxiredoxin-5, mitochondrial	**0.72**	**3.70E-06**	0.26	1.00E+00	**1.37**	**3.12E-11**	**1.14**	**5.95E-08**	**0.65**	**1.78E-09**	**0.88**	**9.31E-06**	**2.28**	**7.60E-12**	**2.02**	**1.10E-11**	**1.14**	**3.11E-11**
Q9JHU4	Dync1h1	Cytoplasmic dynein 1 heavy chain 1	-0.33	6.28E-01	-0.15	1.00E+00	**-0.87**	**4.27E-03**	-0.20	1.00E+00	-0.54	3.41E-01	-0.06	1.00E+00	**-1.11**	**2.08E-04**	**-0.97**	**3.05E-03**	-0.91	2.03E-02
P63005	Pafah1b1	Platelet-activating factor acetylhydrolase IB subunit alpha	0.37	8.76E-01	-0.14	1.00E+00	-0.06	1.00E+00	-0.41	1.10E-01	-0.43	6.17E-01	-0.27	8.04E-01	**-0.57**	**8.10E-03**	-0.43	1.15E-01	-0.16	1.00E+00
Q9QUI0	Rhoa	Transforming protein RhoA	**-0.93**	**5.84E-03**	-0.26	1.00E+00	**-2.22**	**6.54E-07**	-0.59	6.51E-02	-1.28	1.22E-01	-0.33	9.06E-01	**-1.27**	**7.48E-05**	**-1.01**	**6.75E-03**	-0.67	3.77E-01
Q9WVA3	Bub3	Mitotic checkpoint protein BUB3	-0.10	1.00E+00	-0.24	6.56E-01	-0.48	6.41E-02	**-0.65**	**9.57E-04**	-0.38	3.56E-01	-0.41	2.07E-01	**-2.22**	**3.18E-11**	**-1.98**	**1.20E-08**	**-1.57**	**2.90E-04**
Q9CQV8	Ywhab	14-3-3 protein beta/alpha	0.17	1.00E+00	-0.37	7.70E-02	-0.11	9.79E-01	**-0.67**	**2.85E-04**	-0.28	7.38E-01	-0.30	6.09E-01	**-1.70**	**1.71E-10**	**-1.33**	**2.75E-06**	**-1.03**	**4.41E-03**
P62259	Ywhae	14-3-3 protein epsilon	0.19	1.00E+00	**-0.52**	**8.15E-06**	0.27	5.13E-01	**-0.82**	**3.46E-09**	0.07	1.00E+00	-0.30	2.04E-01	**-1.82**	**7.60E-12**	**-1.31**	**2.65E-09**	**-1.01**	**7.89E-05**
P68510	Ywhah	14-3-3 protein eta	-0.35	3.20E-01	**-0.51**	**1.98E-03**	-0.17	6.84E-01	**-1.13**	**3.26E-08**	0.18	7.64E-01	-0.62	2.73E-02	**-4.16**	**7.60E-12**	**-3.66**	**1.16E-10**	**-3.03**	**5.66E-05**
P68254	Ywhaq	14-3-3 protein theta	-0.47	1.11E-01	**-0.83**	**6.23E-05**	**-0.98**	**1.27E-04**	**-0.96**	**1.04E-05**	-0.52	2.71E-01	-0.13	1.00E+00	**-2.17**	**4.71E-11**	**-1.34**	**1.35E-03**	-1.21	2.08E-02
P63101	Ywhaz	14-3-3 protein zeta/delta	0.07	1.00E+00	**-0.66**	**1.22E-05**	0.02	1.00E+00	**-0.84**	**2.94E-07**	-0.05	9.74E-01	-0.19	1.00E+00	**-1.92**	**7.60E-12**	**-1.27**	**4.77E-06**	**-1.08**	**1.11E-03**
Q02248	Ctnnb1	Catenin beta-1	-0.57	4.84E-02	-0.09	1.00E+00	**-1.75**	**4.17E-07**	**-0.47**	**5.44E-04**	-1.18	1.42E-02	-0.38	2.36E-02	**-1.03**	**1.07E-09**	**-0.94**	**3.93E-08**	-0.55	1.18E-02
P60122	Ruvbl1	RuvB-like 1	-0.39	4.84E-01	-0.46	1.64E-01	**-1.07**	**1.31E-03**	**-0.71**	**8.56E-03**	-0.67	2.38E-01	-0.24	1.00E+00	**-2.83**	**2.26E-09**	**-2.36**	**1.48E-05**	**-2.12**	**1.84E-03**
P50136	Bckdha	2-oxoisovalerate dehydrogenase subunit alpha, mitochondrial	-0.50	1.00E+00	-0.12	1.00E+00	0.10	1.00E+00	0.20	1.00E+00	0.60	1.00E+00	0.32	7.62E-01	**1.34**	**7.60E-12**	**1.46**	**1.10E-11**	**1.14**	**1.06E-10**

Figure S1. Individuals factor map of the RNA sequencing data principal component (PC) analysis shows perfect separation of sample groups.

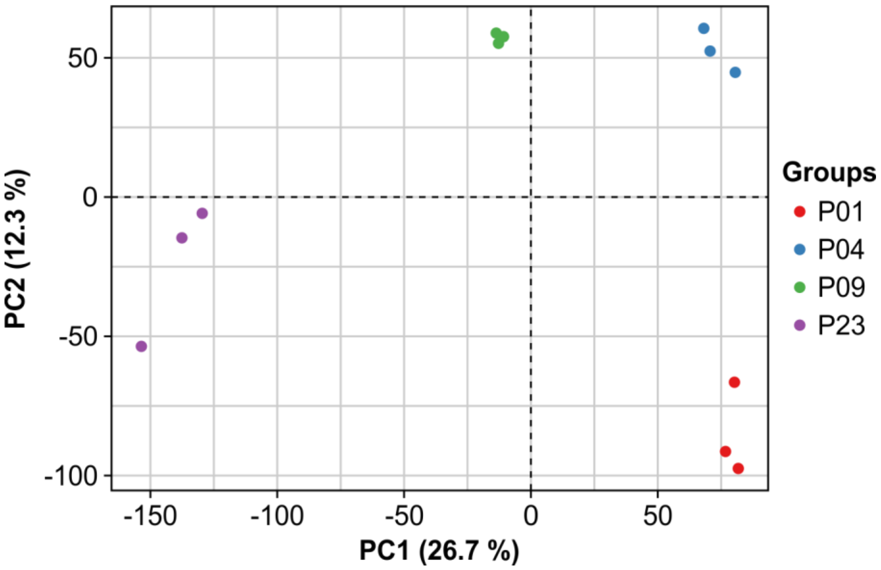

Figure S2. TOP 10 down- and upregulated genes in the postnatal mouse heart.

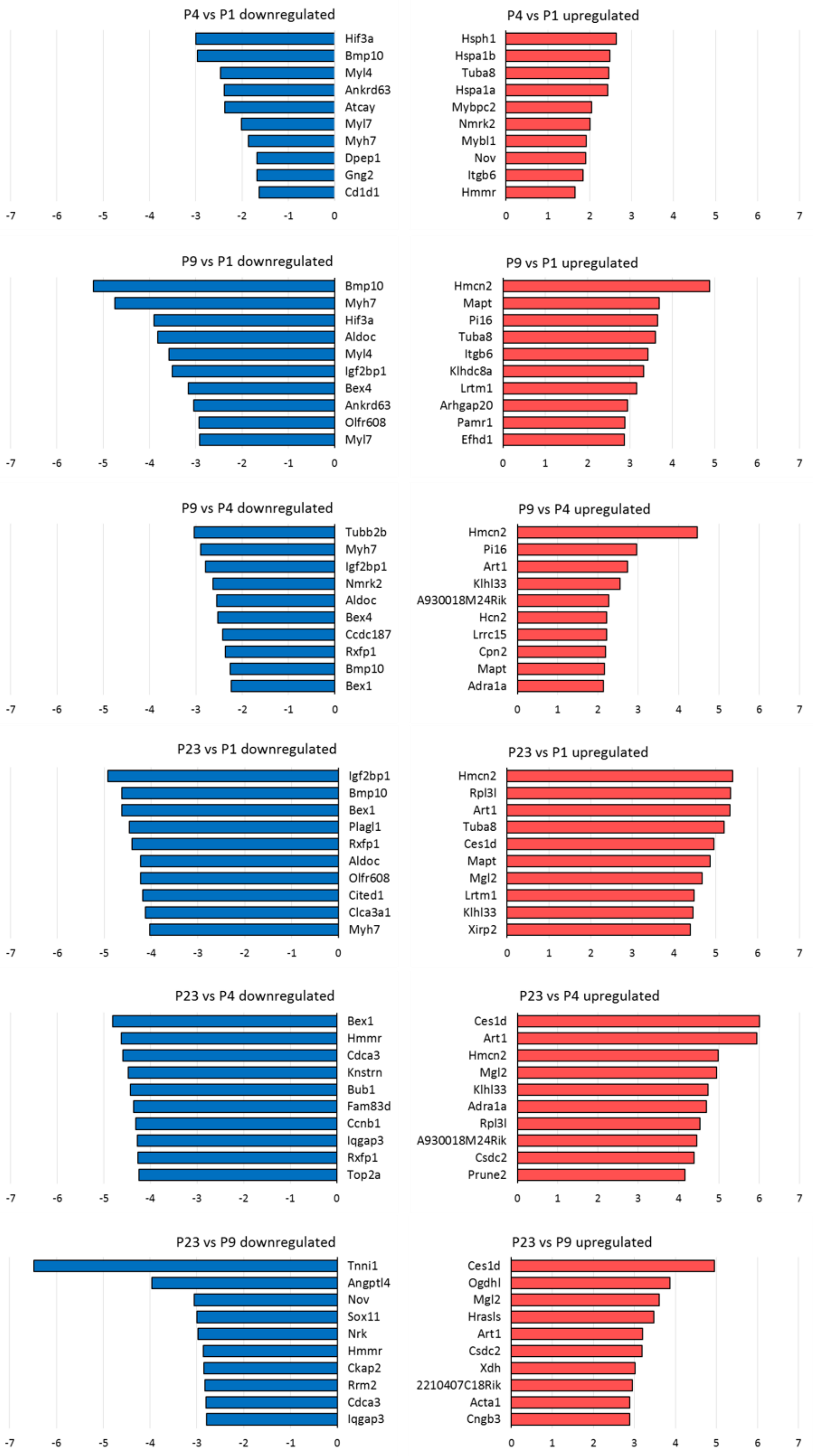

The genes were selected based on fold change among all statistically significantly changes genes ($p < 0.01$) and gene expression changes are presented as log2 fold change. All gene symbol explanations are available in Dataset S1.

Figure S3. Ion channel and control gene expression in the postnatal mouse ventricular tissue.

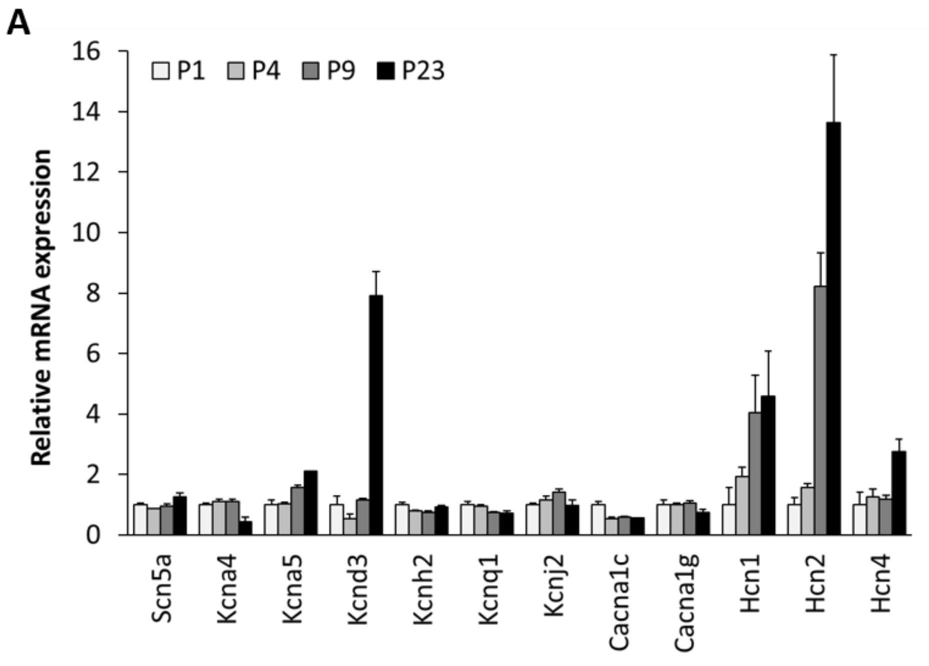

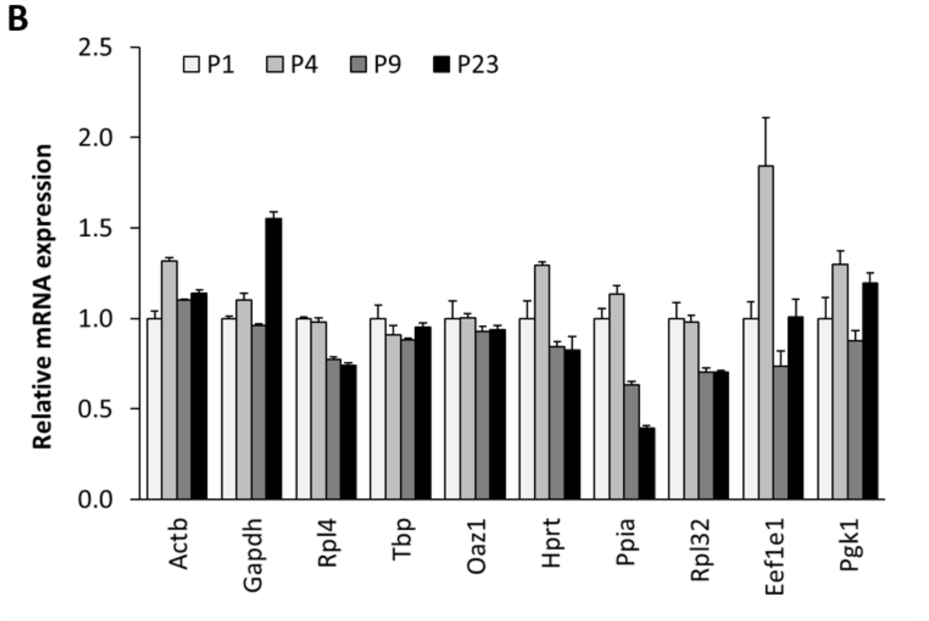

A, Relative expression levels of cardiac ion channels in the mouse ventricular tissue within the postnatal period analysed. **B**, Relative expression levels of control genes in the mouse ventricular tissue within the postnatal period analysed. The data was normalised to P1 and is expressed as mean + SEM from 3 pooled samples (each from 3 hearts). All gene symbol explanations are available in Dataset S1.

Figure S4. Proteomic changes in neonatal mouse hearts.

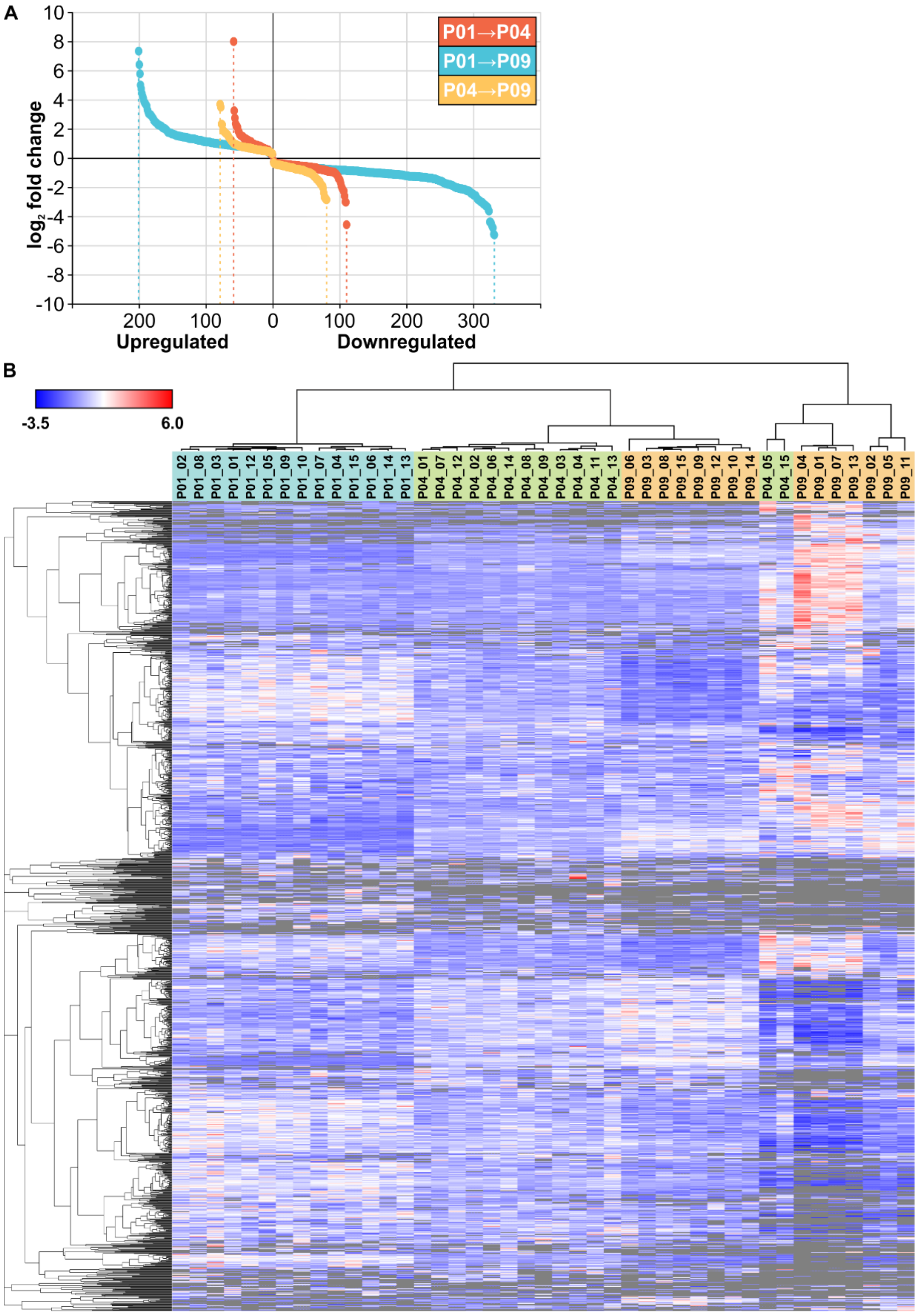

A, The numbers and fold changes of differentially expressed (q < 0.01) proteins in all comparisons between sample groups in sample set 1. **B,** Hierarchical clustering of proteins and samples in sample set 1 shows good separation between sample groups. All proteins detected in > 2/3 samples of at least one sample group are included in the heatmap. Gray color indicates missing values (protein not detected).

Figure S5. Individuals factor maps of the proteomics data principal component (PC) analysis of sample sets 1 (A) and 2 (B).

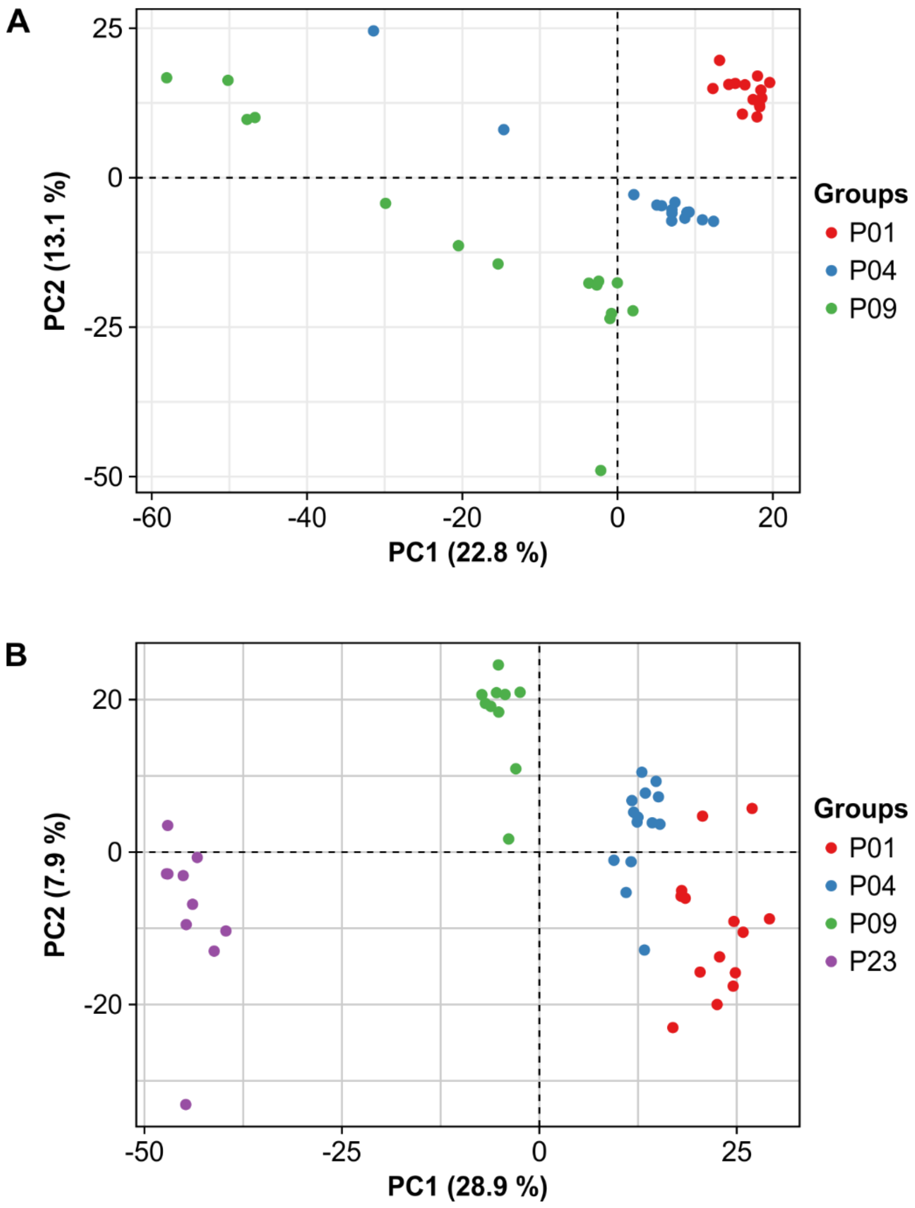

Figure S6. The abundance profiles of all individual transcripts, proteins and metabolites from fuzzy c-means clustering of RNA sequencing, proteomics, and metabolomics data.

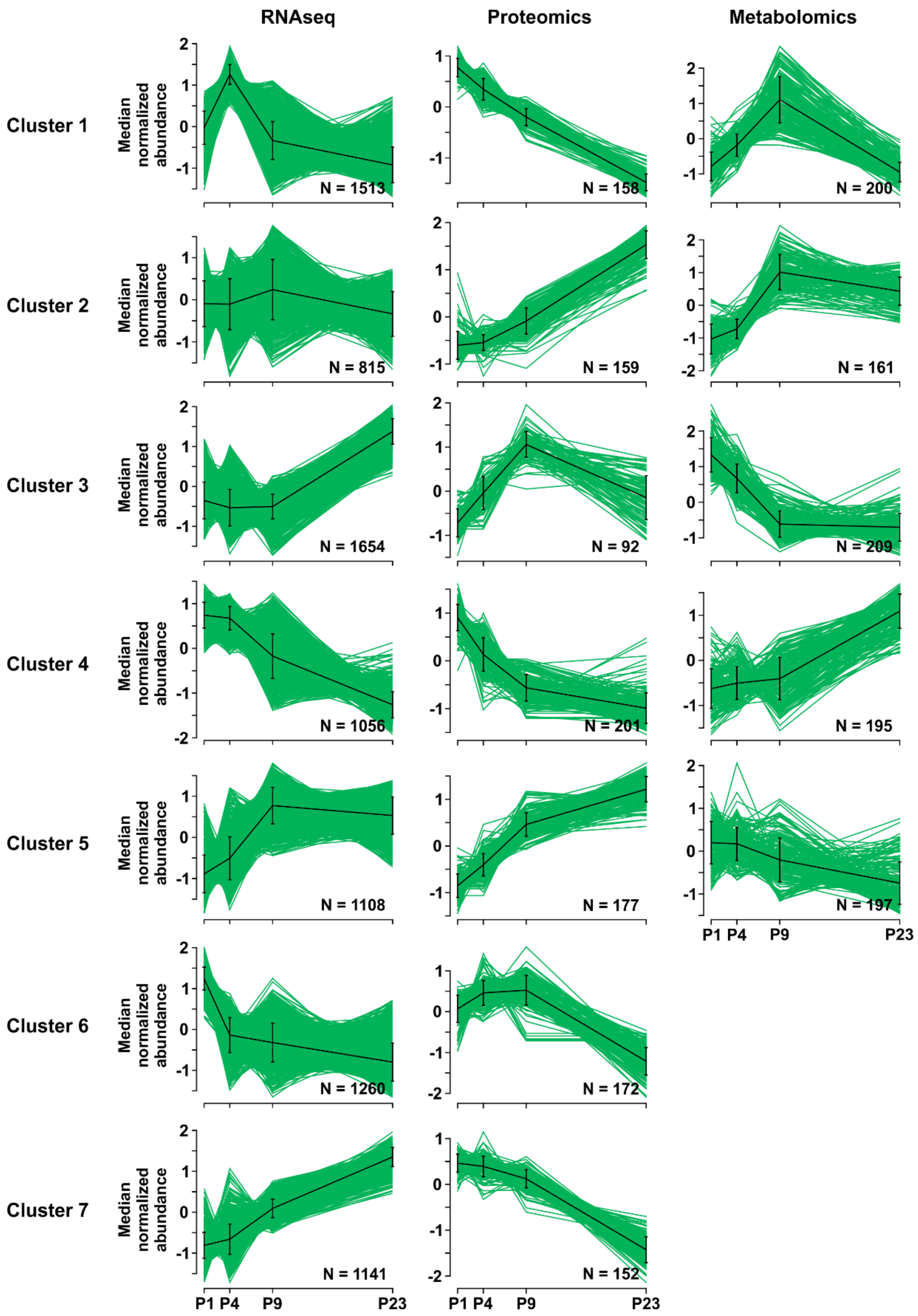

For RNA sequencing and proteomics, respectively, transcripts or proteins detected in > 2/3 samples in at least one sample group were included in the clustering. For metabolomics, both LC-MS/MS and GCxGC-MS data of the metabolites with significantly different abundance in any comparison with LME model were included. The transcripts, proteins, and metabolites belonging to each cluster are presented in Dataset S5.

Figure S7. Fuzzy clustering comparison.

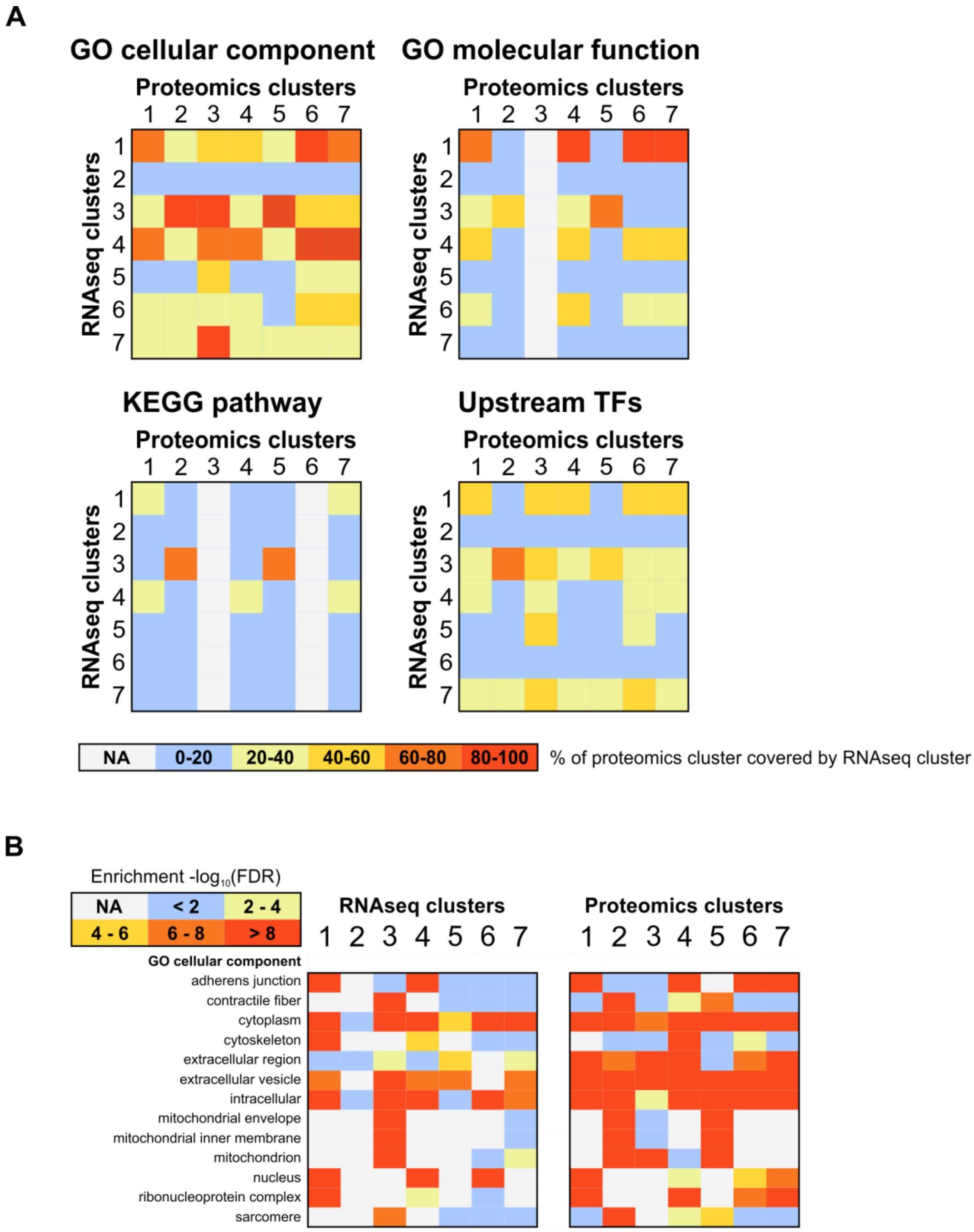

A, The percentage of proteomics fuzzy cluster components covered by RNA sequencing fuzzy cluster components on the levels of GO cellular component, GO molecular function, KEGG pathway (enrichment FDR < 0.01), and potential upstream transcription regulators (p-value < 0.01). **B,** A heatmap of -log10 FDR-values of selected GO cellular component terms in RNAseq and proteomics clusters.

Figure S8. Postnatal changes in glycolysis and gluconeogenesis in the mouse heart.

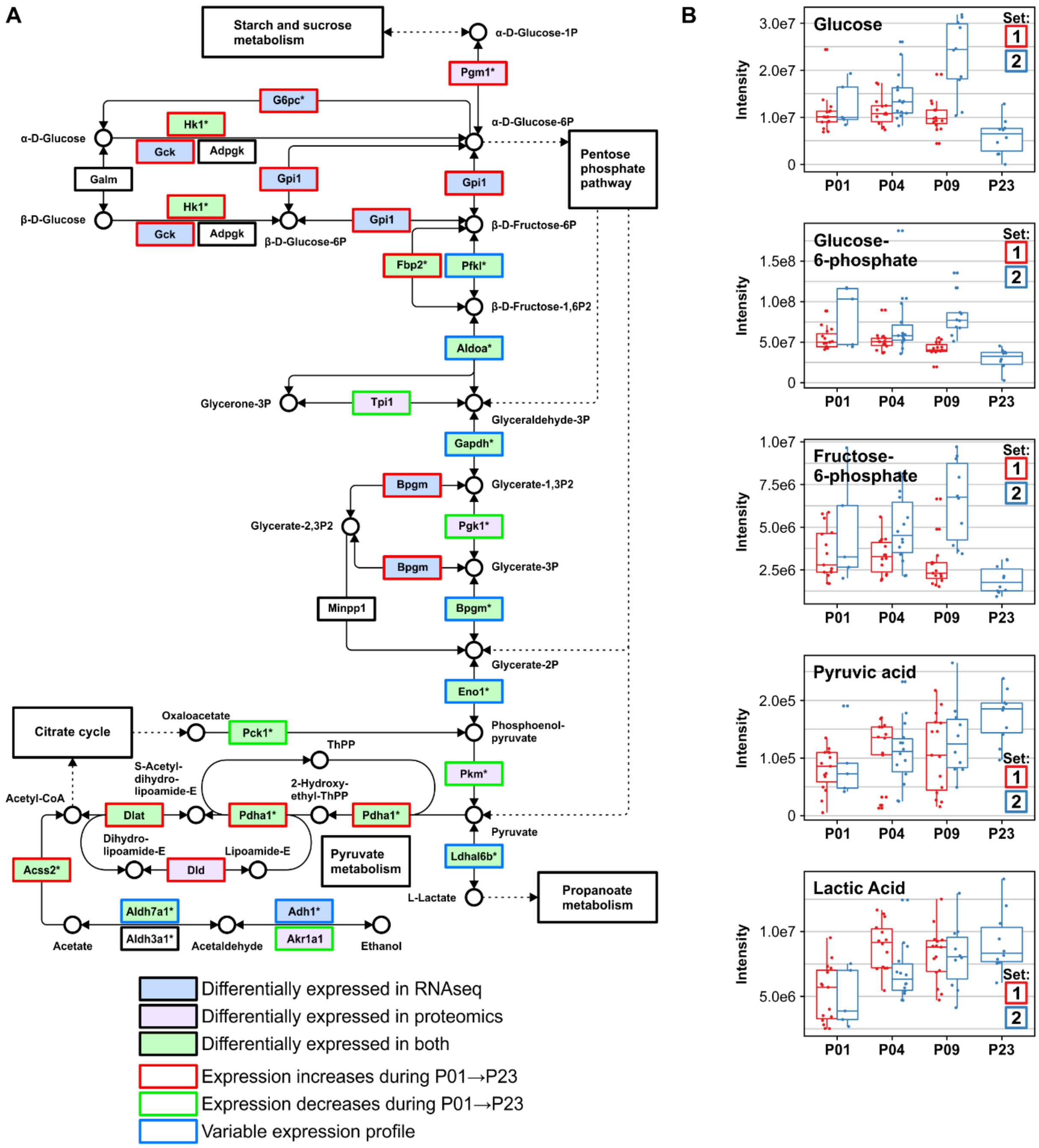

A, Transcriptomic and proteomic changes in the KEGG pathway 'glycolysis and gluconeogenesis' in the early postnatal period. **B**, The abundances of the detected metabolites on the glycolysis and gluconeogenesis pathway. All gene symbol explanations are available in Dataset S1. The KEGG pathway image is modified and reprinted from http://www.genome.jp/kegg/ with permission from the Kyoto Encyclopedia of Genes and Genomes.

Figure S9. Postnatal changes in the pyruvate pathway in mouse heart.

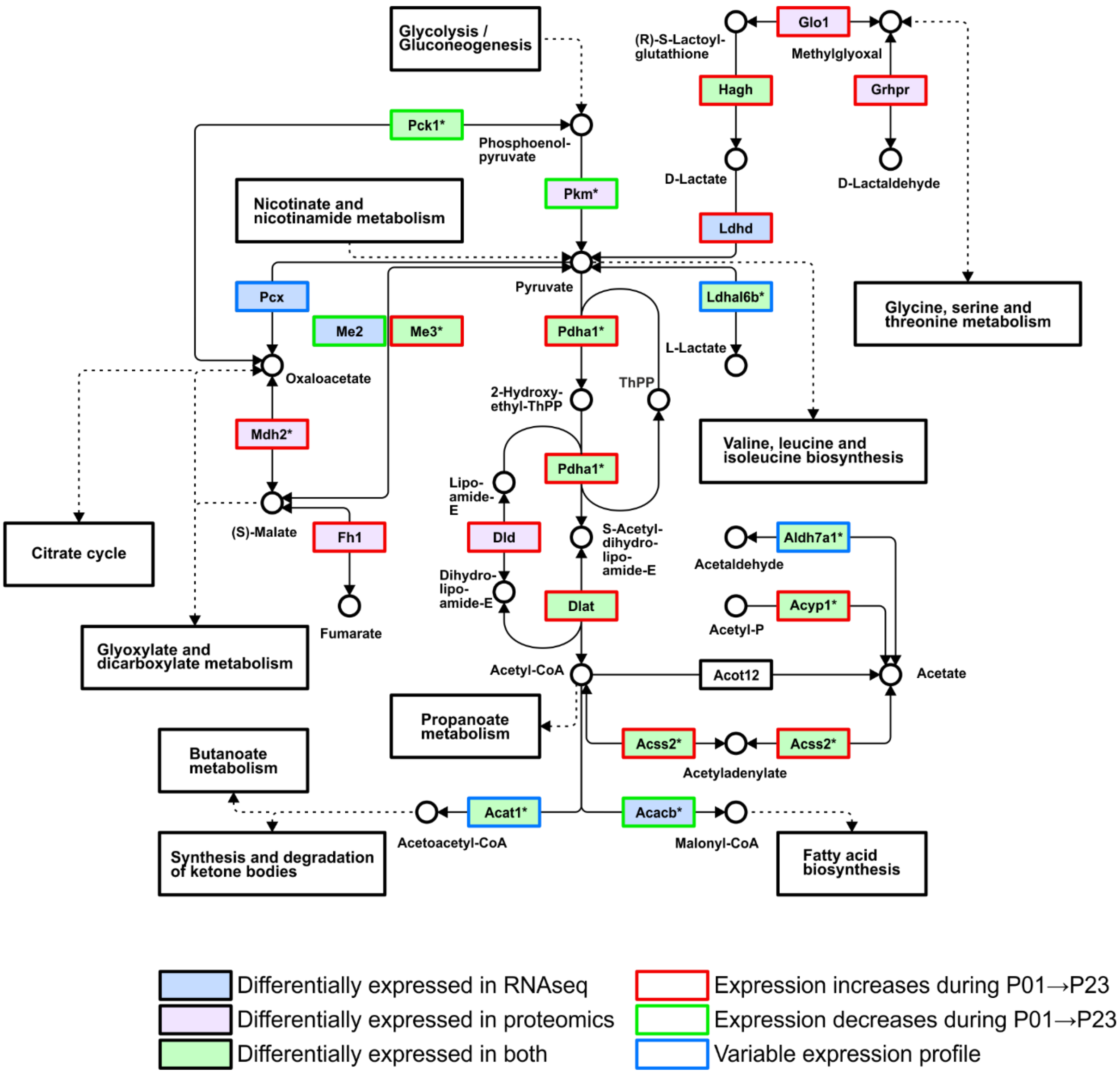

Transcriptomic and proteomic changes in the KEGG pathway 'glycolysis and gluconeogenesis' in the early postnatal period. All gene symbol explanations are available in Dataset S1. The KEGG pathway image is modified and reprinted from http://www.genome.jp/kegg/ with permission from the Kyoto Encyclopedia of Genes and Genomes.

Figure S10. Postnatal changes in fatty acid degradation in mouse heart.

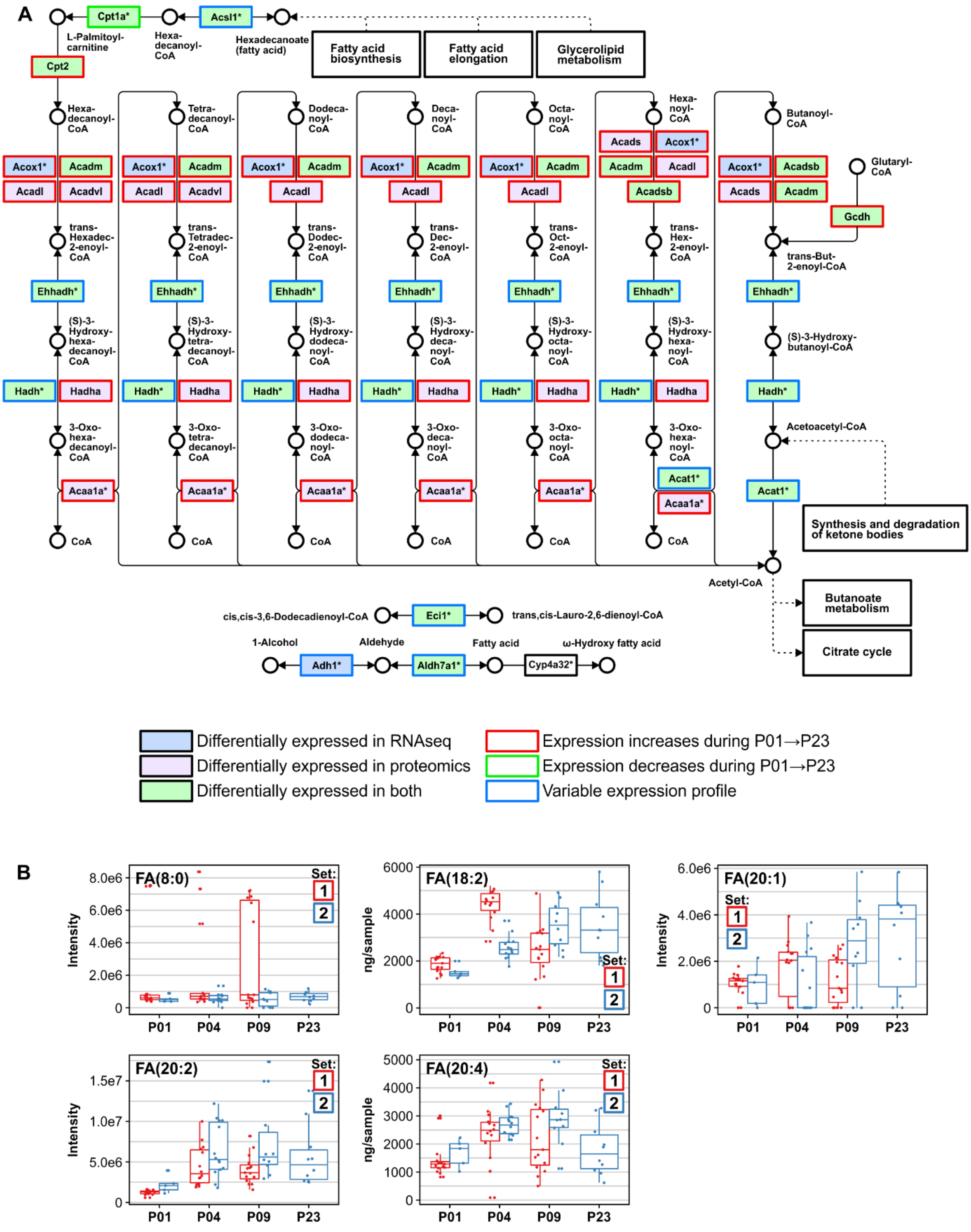

A, Transcriptomic and proteomic changes in the KEGG pathway 'fatty acid degradation'. **B**, The abundances (intensity) or concentrations of selected fatty acids (FAs). All gene symbol explanations are available in Dataset S1. The KEGG pathway image is modified and reprinted from http://www.genome.jp/kegg/ with permission from the Kyoto Encyclopedia of Genes and Genomes.
